# Supplementary material for: Drug-resistant EGFR mutations promote lung cancer by stabilizing interfaces in ligand-free kinase-active EGFR oligomers
Source: Nat Commun. 2024 Mar 19;15:2130. doi: 10.1038/s41467-024-46284-x (PMC10951324; doi:10.1038/s41467-024-46284-x)
Supplement: Supplementary file 1 — Supplementary Information [file 41467_2024_46284_MOESM1_ESM.pdf]

Supplementary Information

Drug-resistant *EGFR* lung cancer mutations promote lung cancer by stabilizing interfaces in ligand-free kinase-active EGFR oligomers

R. Sumanth Iyer, Sarah R. Needham, Ioannis Galdadas, Benjamin M. Davis, Selene K. Roberts, Rico C.H. Man, Laura C. Zanetti-Domingues, David T. Clarke, Gilbert O. Fruhwirth, Peter J. Parker, Daniel J. Rolfe, Francesco L. Gervasio, and Marisa L. Martin-Fernandez

Contents:

Supplementary Fig. 1: FLImP resolution test and controls using single particle tracking (SPT), confocal microscopy, and photobleaching correlation spectroscopy, related to main text Fig. 3 and Fig. 4.

Supplementary Fig. 2: Effects of tether disrupting mutations on the $H2H^{ect}{}_{dimer}$ sub-unit and $B2B^{ect}{}_{dimer}$ sub-unit seen in the MD simulations, related to main text Fig. 5.

Supplementary Fig. 3: Experimental results for G564P-EGFR and Lzip3A-EGFR, related to main text Fig. 5 and Fig. 6.

Supplementary Fig. 4: Steps for construction of the ligand-free EGFR hetero-oligomer, related to main text Fig. 7.

Supplementary Fig. 5: Experimental 2D FLImP data and analysis for T766M-EGFR, related to main text Fig. 7.

Supplementary Fig. 6: The H566F and Lzip3S mutations interfere with T766M-EGFR oligomer formation, related to main text Fig. 7.

Supplementary Fig. 7: Modelling and simulations of the $Bb2Bb^{kin}{}_{dimer}$ sub-unit. Quantification Western Blot from main text Fig. 6d. Western Blot and its quantification of WT and T766M phosphorylation in the presence of the H566F and K946E mutations. Related to main text Fig. 8.

Supplementary Fig. 8: Modelling and simulations of the $Bb2Bb^{kin}{}_{dimer}$ sub-unit in Exon 20 mutations. Comparison between FLImP distributions for InsNPG and WT + Erlotinib. Related to main text Fig. 8.

Supplementary Fig. 9. Modelling of a “zig-zag” tetramer and mutations that strengthen the $S2S^{kin}{}_{dimer}$, related to main text Fig. 9.

Supplementary Fig. 10. Growth controls in Ba/F3 cells and data supplementing the mice study, related to main text Fig. 10.

Supplementary Table 1: Reliability and reproducibility checklist for molecular dynamics simulations.

Supplementary Table 2: System details of the simulated systems.

Supplementary Table 3: Separations predicted by the model and those satisfied by mutations and treatments, highlighting changes from cancer mutations.

Supplementary Table 4: Primers used for Site-Directed Mutagenesis or to PCR amplify EGFR.

Supplementary Table 5: Parameters of the 1-way ANOVA analysis with Tukey’s multiple comparison correction performed. Number of comparisons per family=6, α=0.05, related to main text Fig. 10.

Supplementary Note 1: MD simulations and structural modelling.

Supplementary Note 2: FLImP automated data acquisition.

Supplementary Note 3: FLImP data analysis.

Uncropped Western Blots: Relevant to Fig. S1f, Fig. S3e, Fig. S7c.

Supplementary Figure 1:

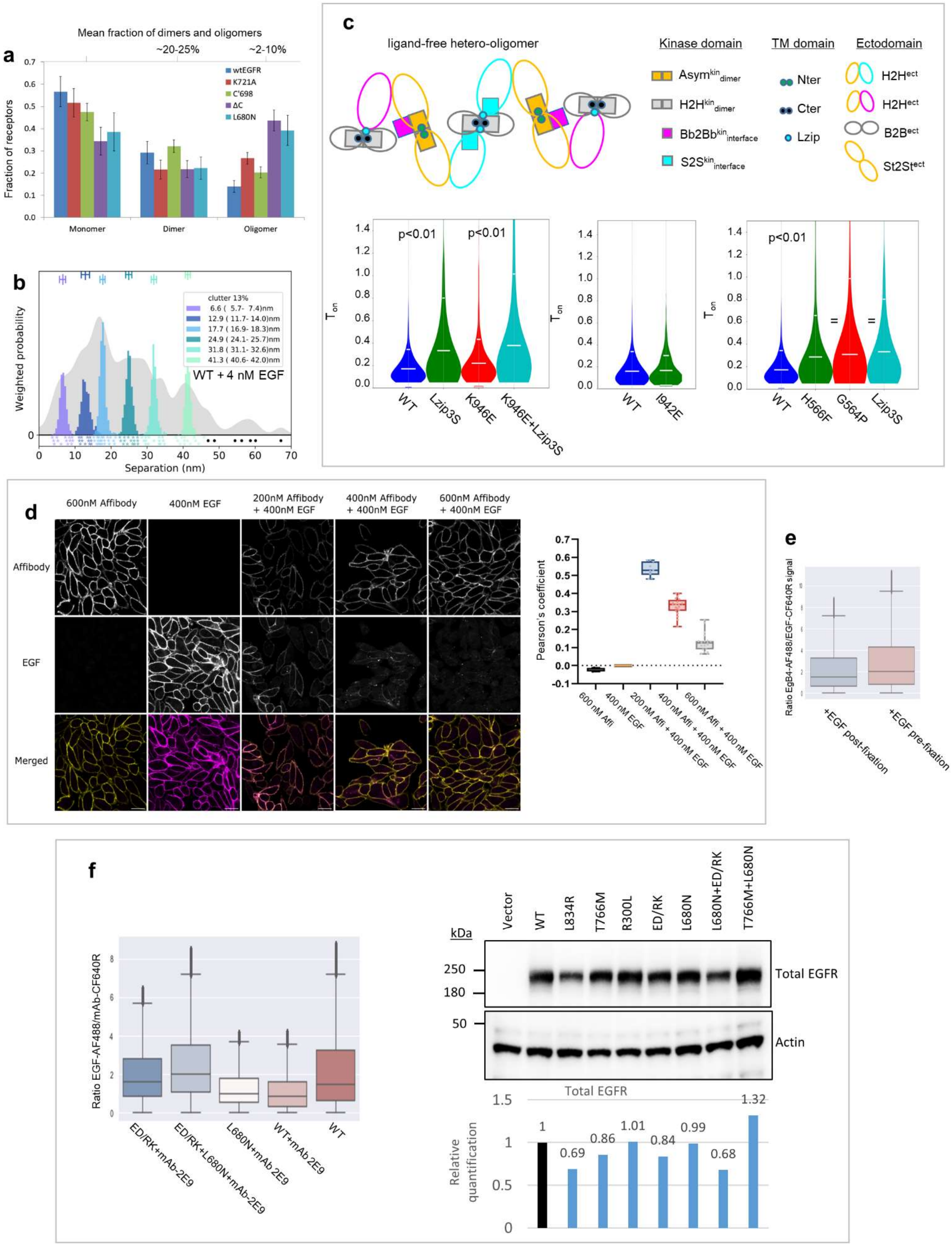

Supplementary Fig. 1. FLImP resolution test and controls using single particle tracking (SPT), confocal microscopy and photobleaching correlation spectroscopy related to main text Fig.3, and Fig. 4.

a Molecular-normalized fraction of receptors in oligomer species measured in CHO cells expressing ~100,000 receptor copies per cell. Cells were treated with 100 nM Alexa 488-Affibody, and the oligomer fraction was determined by photobleaching imaging correlation spectroscopy, as previously shown[1]. The average fraction of dimers and oligomers corresponding to these data is also shown. The lower and upper limits in the fraction of oligomers are estimations depending on the size of oligomers. (We considered sizes from tetramers up to oligomers with 20 protomers). N= 3, error bars = standard deviation (SD).

b FLImP analysis of 100 separation probability distributions between EGF-CF640R pairs on CHO cells labelled with 4 nM EGF-CF640R like in: Sum of posteriors of individual separations between fluorophores (gray background) and abundance-weighted probability distributions of individual components of decomposed separation distribution (colored peaks). The peak positions are indistinguishable to those in[2] but the resolution has been halved using the new analysis. Black dots represent clutter.

c Top, Model of ligand-free EGFR oligomers derived from FLImP data. The proposed oligomer is assembled from 10 interfaces of different strengths, i.e., that have different buried surface areas in protein-protein complexes. Bottom, use of two-color SPT of cell surface receptors in live transfected CHO cells[1] to check for artifacts due to cell fixation. Cells were labeled with a 1:1 mixture of anti-EGFR Affibody-Alexa 488 and Affibody-CF640R. Two color SPT reports the incidence of pairwise particle interactions and their duration (T_{on}). T_{on} therefore reflects the combined strength of the interactions holding the oligomer together, and thus should reflect changes in the dynamics of oligomer disassembly when mutations are introduced. Data was acquired over at least 30 cells per condition, acquired over at least 3 independent biological replicates. Although it is hard to predict how the breaking of a large/complex oligomer in two portions might translate in changes in T_{on}, a few assumptions appear reasonable: Bottom left panel, we would expect that the Lzip3S mutations will increase T_{on} when Lzip contacts participate in oligomer assembly. The rationale is that oligomers will more often break via the weakest assembling interaction, in this case, transmembrane contacts. Among the latter, the Lzip interface is not integral to any given dimer species. We therefore speculated that, in our time observation window, which is from zero to a maximum dictated by the lifetime of the fluorophores employed, the observed pairwise T_{on} should be biased towards the short duration of the Lzip interfaces. Thus, if the Lzip3S mutations are introduced, the resultant mutant oligomers will have to break via stronger interfaces, e.g., between ectodomains or kinase domains, that would be predicted to last longer. The increase in T_{on} observed when the Lzip3S mutations are introduced is consistent with our speculations. Bottom middle panel, a second assumption safe to make refers to the I942E mutation, which strengthens the $S2S^{kin}_{interface}$. Because I942E-EGFR oligomers will still break via weak Lzip interfaces, I942E should not increase T_{on}, as shown by the data. Bottom right panel*,* A third assumption safe to make is that since the tether-disrupting mutations and the Lzip3S mutations have a similar effect on the conformation of the $H2H^{ect}/2x^{kin}_{monomers}$ sub-unit, we would expect that the T_{on} values for these mutations would be similar among each other, and in turn different to WT-EGFR. This is what we found. The agreement between these predictions and the two-color SPT results backs previous results[2] in which we could not detect that the cell fixation procedure introduced artefacts.

d Left, representative confocal images of cells labeled with Affibody-CF640R (top) at 4°C before fixation and EGF-Alexa488 (middle) after fixation and merged (bottom panels). Right, Numerical analysis of the overlaid colocalization images. A decrease in the Pearson colocalization coefficient clearly shows that Affibody and EGF compete for the same EGFR binding sites.

e Comparison of the degree of labeling with EGF-CF640R before fixation at 4°C and after cell fixation. Results show the fixative does not impede the binding of EGF-CF640R after cells were fixed. Ratios were calculated pixel-wise on data acquired over at least 30 cells. The datapoints were 2288849 for post-fixation and 1500343 for pre-fixation. The whiskers encompass 1.5 IQRs of the lower and upper quartile. Outliers are represented as black dots.

f Left, EGF-binding comparisons on different cell lines pre-treated with 200 nM mAb-2E9 prior to fixation and EGF labeling: A previous quantitative ^{125}I-EGF binding analysis proposed that Erlotinib promotes inside-out high affinity EGF binding.[3] In addition, Erlotinib promotes the $Asym^{kin}_{dimer}$ sub-unit[3]. Together, these results

suggest that the St2St^{ect}/Asym$^{kin}_{dimer}$ sub-unit binds EGF with high affinity. To test this possibility, we labeled cells with 200 nM mAb-2E9 tagged with Alexa Fluor 488, which selects for high affinity EGF binding[4], then fixed the cells and then added 200 nM EGF-CF640R, measuring the degree of EGF binding still possible after mAb-2E9 treatment by ratioing the two intensity values. The results show that mAb-2E9 blocks as much EGF binding to cells expressing WT-EGFR as to cells expressing the L680N-EGFR mutant. L680N is an N-lobe mutation of the kinase that inhibits the St2St^{ect}/Asym$^{kin}_{dimer}$ sub-unit. In contrast, binding is not reduced when the St2St^{ect}/Asym$^{kin}_{dimer}$ sub-unit is present but the B2B^{ect}/H2H$^{kin}_{dimer}$ sub-unit has been inhibited by the ED/RK mutation. Interestingly, the results indicate that mAb-2E9 does not block the binding of EGF to the H2H^{ect}/2X$^{kin}_{monomers}$ sub-unit. Ratios were calculated pixel-wise on data acquired over at least 30 cells, with n> 100,000 for individual datapoints. The whiskers encompass 1.5 IQRs of the lower and upper quartile. Outliers are represented as black dots. Top-Right, Western blot showing that the stably expressing CHO cells lines used express comparable levels of WT-EGFR and receptor mutants to experiment in (*Left*). Bottom-Right, Western blots from whole cell lysates of untreated CHO stable cell lines expressing different EGFR mutants, normalized for total protein content and probed with Anti-EGFR, show a comparable expression level when comparing cells expressing WT-EGFR, ED/RK-EGFR and L680N-EGFR. Quantification by densitometry of blot shown confirms this. The blot shown is representative of n=3. Data for the box plots in panels e) and f) is available on Zenodo as DOI[5]. All other source data are provided as a source data file.

Supplementary Figure 2:

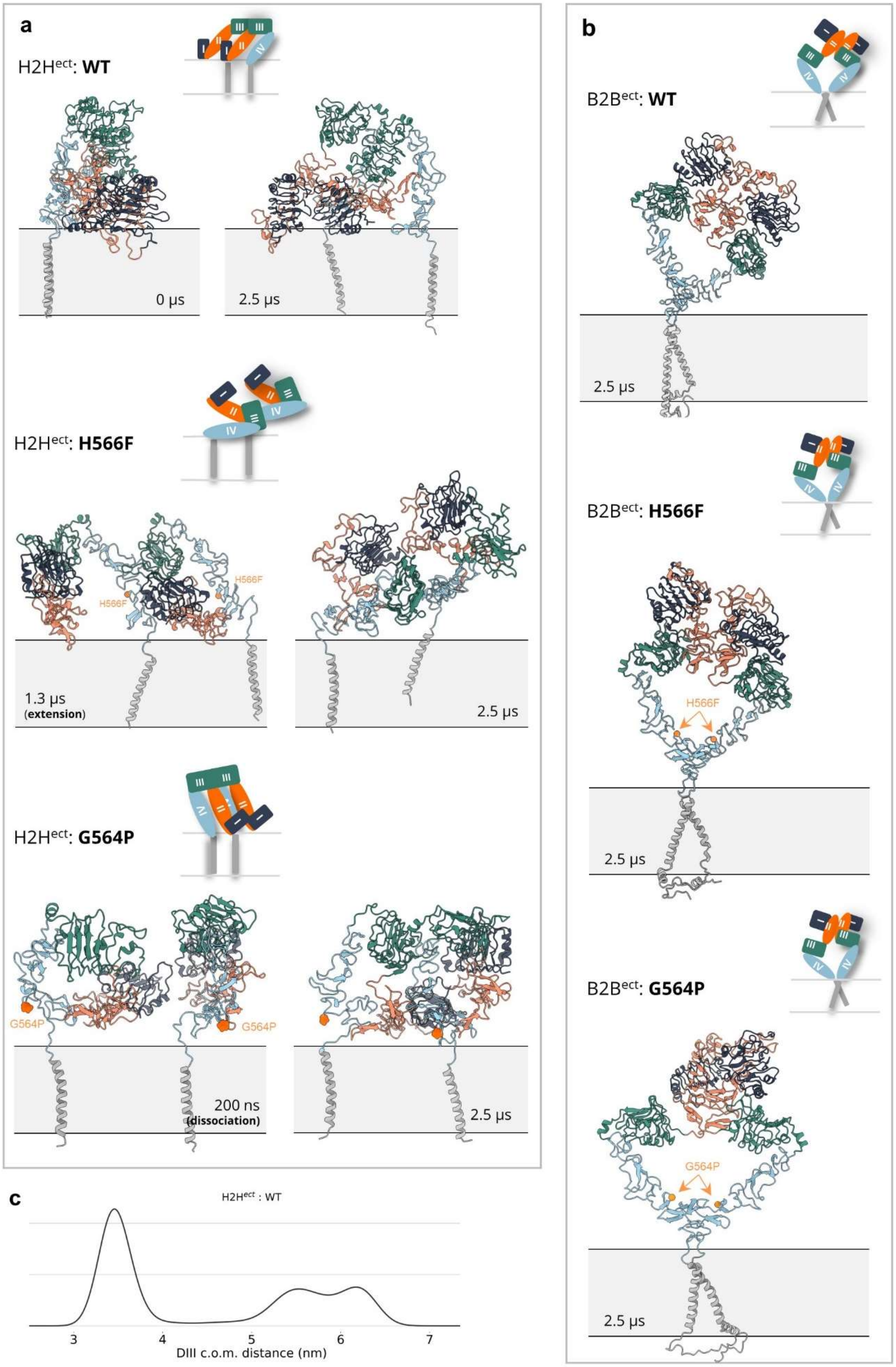

Supplementary Fig. 2. Effects of tether disrupting mutations on the $H2H^{ect}_{dimer}$ sub-unit and $B2B^{ect}_{dimer}$ sub-unit seen in the MD simulations, related to main text Fig. 5.

a Snapshots extracted from different time points of a 2.5 µs long simulation of the $H2H^{ect}_{dimer}$ sub-unit and their TM domains in the lipid bilayer. The glycans bound to the ectodomain of each monomer are not shown for clarity. A cartoon representation of the dimer in the last frame of the simulation of each variant is drawn. (Explained further in Supplementary Note 1). Top, Over the course of the simulation, the ectodomains (ECDs) of WT proved flexible, transitioning to a state where the ECDs oriented themselves parallel to each other and vertically to the membrane. Middle, Early in the simulation, the H566F mutation removes the hydrogen bonds that H566 forms with the backbone and sidechains of T250, Y251, and Q252 from the WT DII-DIV interface. The loss of these interactions compromises the tethered conformation, and the H566F mutant transiently adopts an open conformation. Despite the transient opening, the H566F mutant is found in an $H2H^{ect}_{dimer}$ sub-unit in most parts of the simulation. Unlike the WT, though, in this tethered conformation, DI (purple) moves away from the membrane and closer to DIII (green), making the EGF binding site less accessible in both monomers. The destabilization of the DII-DIV interface upon mutation is expected to increase the population of extended conformations that are supposed to bind EGF with higher affinity. Bottom, The introduction of a Pro through the G564P mutation significantly alters the backbone configuration in the region of the DIV (blue), which interacts with the dimerization arm on DII (orange), thus, disrupting several intramolecular interactions that lead to the dissociation of the two monomers.

b Snapshots extracted from the last frame of a 2.5 µs long simulation of the $B2B^{ect}_{dimer}$ sub-unit and their TM domains in the lipid bilayer. The glycans bound to the ectodomain of each monomer are not shown for clarity. (Explained further in Supplementary Note 1). A cartoon representation of the dimer in the last frame of the simulation of each variant is drawn. Although both monomers of the $H2H^{ect}_{dimer}$ H566F and G564P underwent larger-scale DII oscillations that triggered untethering, the monomers of the $B2B^{ect}_{dimer}$ of both variants stay in contact, and the dimers are stable over the course of the 2.5 µs long simulations.

c Distribution of the center of mass (c.o.m.) distance of the DIII domains where the fluorescent affibody binds over the course of the 2.5 µs long MD simulation of the $H2H^{ect}$ of the WT-EGFR.

Supplementary Figure 3:

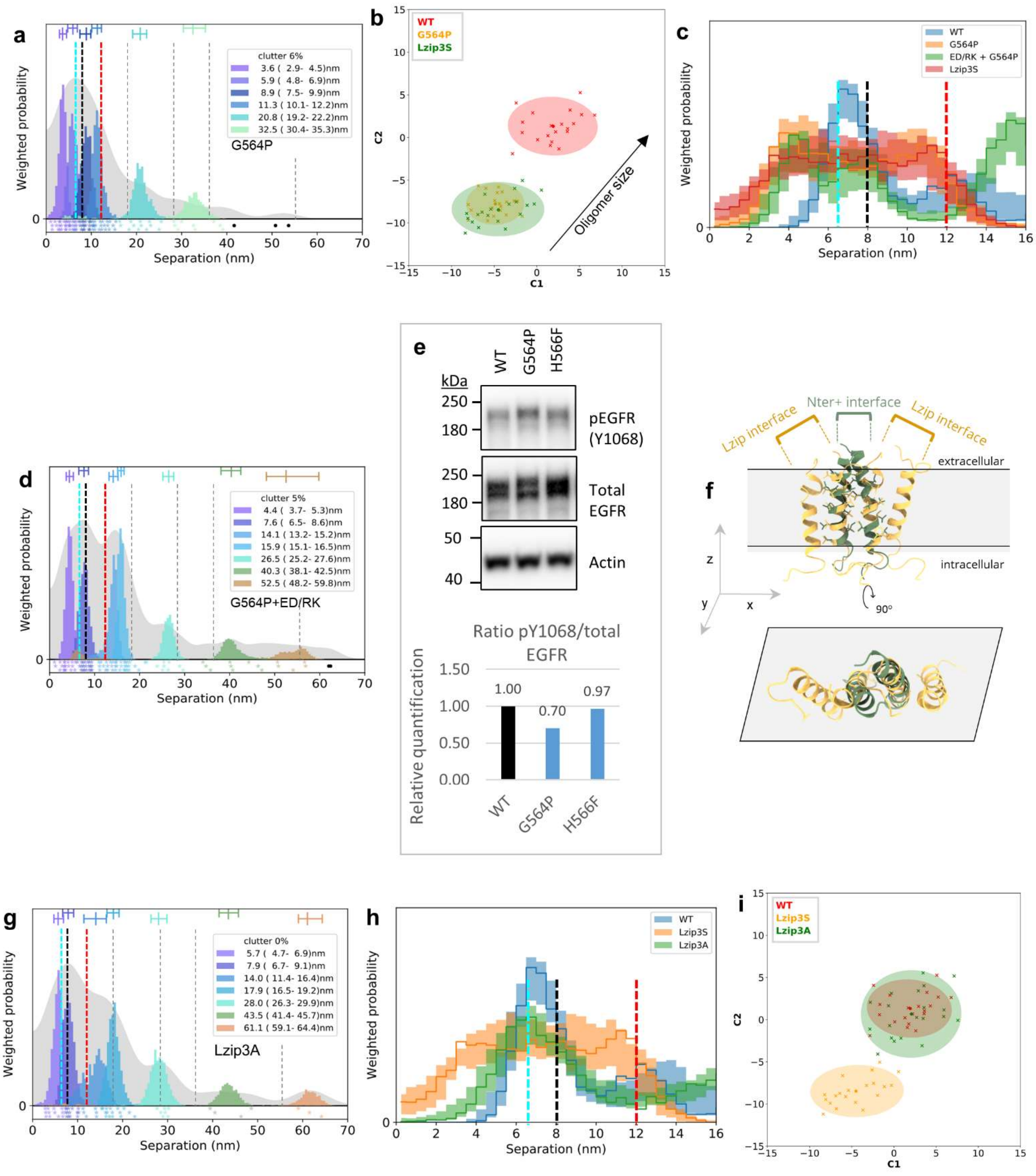

Supplementary Fig. 3. Experimental results for G564P-EGFR and Lzip3A-EGFR, related to main text Fig. 5 and Fig. 6.

a, d, g FLImP analysis of 100 separation probability distributions between Affibody-CF640R pairs in the conditions indicated: Sum of posteriors of individual separations between fluorophores (gray background) and abundance-weighted probability distributions of individual components of decomposed separation distribution (colored peaks). Plot legend and bars above colored component distributions give the median

and most-compact 68% confidence interval for each. Legend also gives median proportion of measurements assigned to clutter. The median peak positions marked by dashed lines are those of WT-EGFR.

b, i Wasserstein MDS analysis of the FLImP decompositions for the conditions in the inset. The similarities or dissimilarities between the 21 separation sets associated to different conditions (one main FLImP decomposition plus 20 Bootstrapped with resampling decompositions) are compared; in this case, those associated to the separation sets for G564P-EGFR, Lzip3S-EGFR, Lzip 3A-EGFR, and WT-EGFR. The axes in the plot are Component 1 (C1) and Component 2 (C2). C1 represents the dimension that captures the largest amount of variance in the data, while C2 represents the second-largest amount of variance that is orthogonal to C1. The centers of the 95% confidence ellipses mark the mean positions of the main FLImP decompositions. The crosses mark the positions of individual bootstrapped separation sets.

c, h Comparisons between decomposed separation probability distributions between FLImP datasets. The continuous lines show the marginalized separation posterior, i.e., the sum of the abundance-weighted peaks, for each condition in the inset. The fluctuations around each continuous line arise from variations derived from FLImP decompositions for 20 bootstrap-resampled datasets to assess errors due to finite number of measurements. Note the similarity between the data for G564P-EGFR and Lzip3S-EGFR in **c** (orange and red lines). We investigated whether the increase in separation density at ~10-12 nm was related to the $B2B^{ect}/H2H^{kin}_{dimer}$ sub-unit by combining the G564P and ED/RK mutations (**c**, green line). Results show that inhibiting the latter decreases the density at this interval. The median peak positions marked by dashed lines are those of WT-EGFR.

e Top, Western blot comparing WT, G564P and H566F EGFR phosphorylation in C-terminal residue Y1086 in transfected CHO cells. Bottom, Quantification by densitometry of blot shown. The blot shown is representative of n=3.

f ***Top***, A speculative tetramer (yellow) formed by two monomers interacting through Lzip contacts with one Nter+ interface. A model of the Nter+ dimer (green) is placed on top of the tetramer showing that the residues that participate in the Nter+ interface are distinct from the Lzip. Bottom, orthogonal projection on xy-plane. Source data are provided as a source data file.

Supplementary Figure 4:

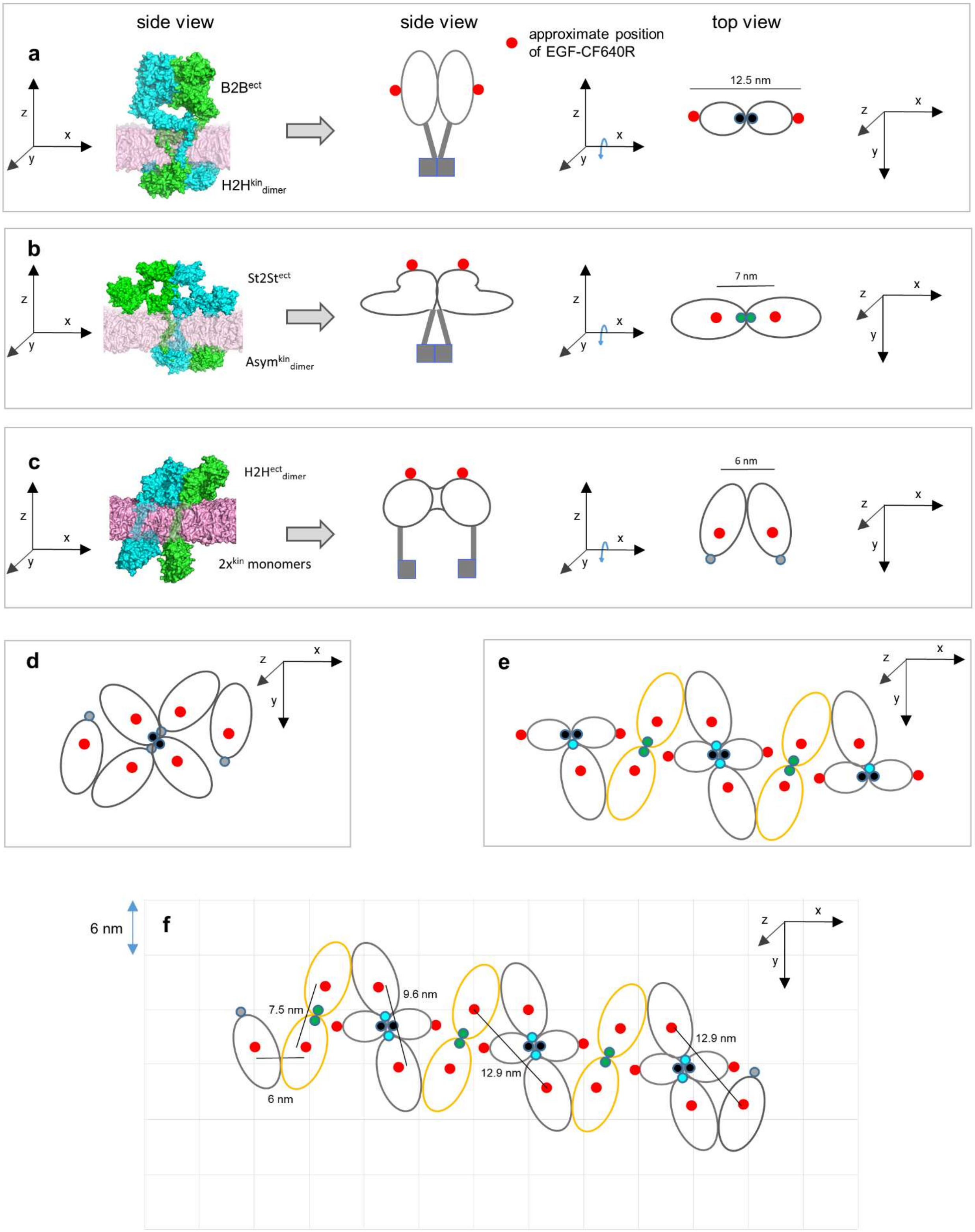

Supplementary Fig. 4. Steps for construction of the ligand-free EGFR hetero-oligomer, related to main text Fig. 7.

a $B2B^{ect}/H2H^{kin}_{dimer}$ sub-unit[6] is simplified into a cartoon of approximate dimensions. **b** is the $St2St^{ect}/Asym^{kin}_{dimer}$ sub-unit[1] and **c** is the $H2H^{ect}/2x^{kin}_{monomers}$ sub-unit[1]. The red dots mark the positions where we would expect the fluorescent EGF derivative (EGF-CF640R) to bind after cells are fixed. The blue dots mark Lzip interfaces between a transmembrane monomer and a Cter dimer (black dots). The green dots represent Nter transmembrane dimers. The cartoon model of the $B2B^{ect}/H2H^{kin}_{dimer}$ sub-unit is then rotated around the x-axis. A top view projection of the dimer model is shown. The grey dots represent transmembrane monomers.

d The attempt to connect $H2H^{ect}/2x^{kin}_{monomers}$ sub-unit and the $St2St^{ect}/Asym^{kin}_{dimer}$ sub-unit resulted in steric clashes that prevented the formation of hetero-oligomers as large as those reported by the data.

e, **f** $Hetero^{conf}$-oligomers large enough to match the data could be formed by connecting a $H2H^{ect}/2x^{kin}_{monomers}$ sub-unit and the $B2B^{ect}/H2H^{kin}_{dimer}$ sub-unit, as shown. The model predicts the distances experimentally found by 1D and 2D FLImP. Note that the intracellular portion is not shown for simplicity.

Supplementary Figure 5:

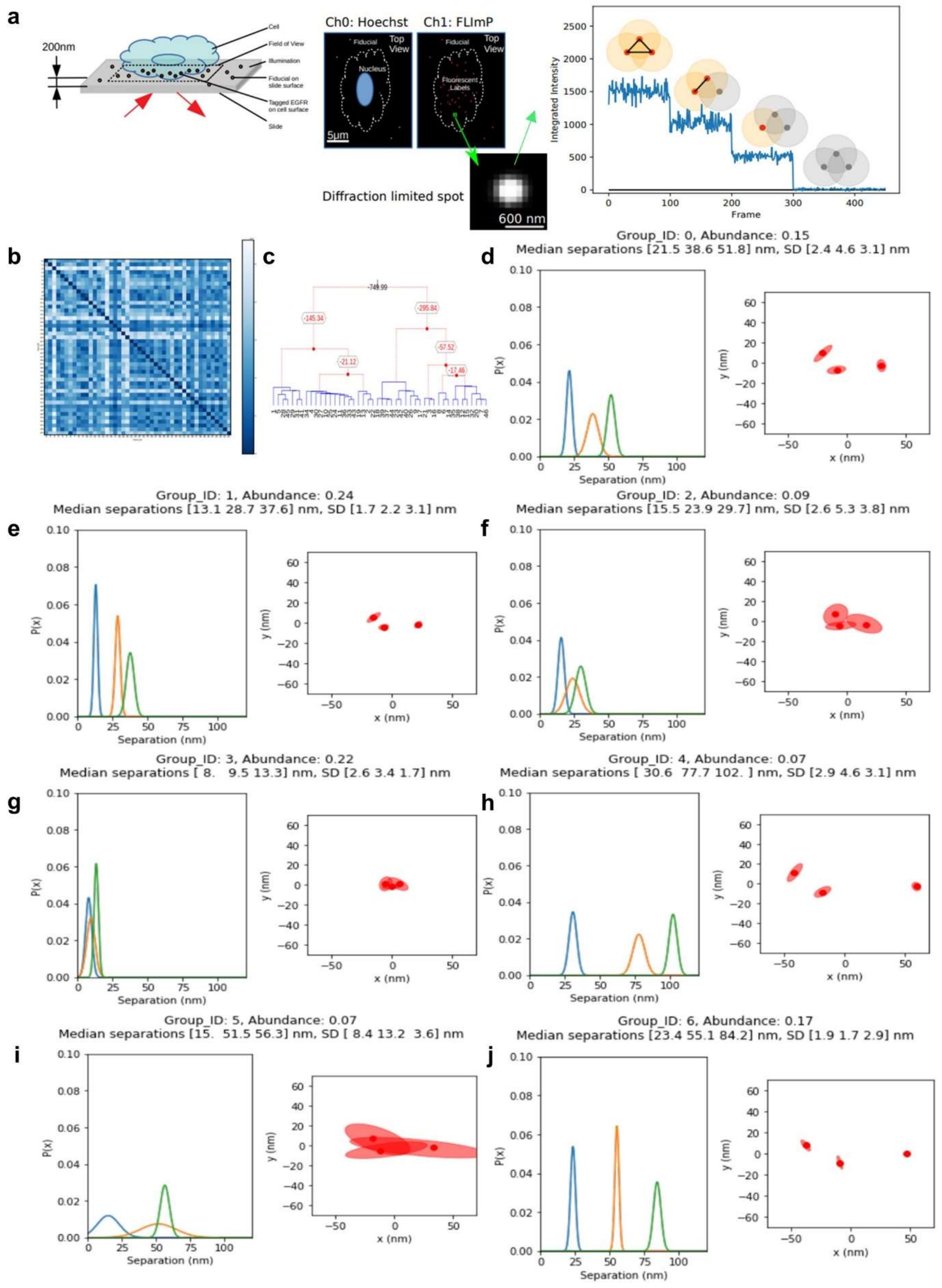

Supplementary Fig. 5. Experimental 2D FLImP data and analysis for T766M-EGFR, related to main text Fig. 7. (Explained further in **Supplementary Note 3,** part 9)**.**

a For 2D FLImP CHO cells were imaged using total internal reflection microscopy. The cells were fixed and labeled with EGF-CF640R for FLImP and Hoechst[7] for cell detection, the latter to verify that data was collected on cells and not glass. Images of labeled cells were acquired, and the intensity of individual spots plotted as a function of time. Spots that decay in three steps (containing at least three fluorophores) were selected for further analysis.

b 2D FLImP imaging returned a population of 46 triangles, providing information about the separations between groups of three fluorescently-labeled locations in the sample of interest. Distances between each pair of 2D-FLImP posteriors were measured using the Wasserstein metric[8], whereby identical 2D-histograms would have a Wasserstein metric of zero (blue) and more different histograms would exhibit larger values (white). As the Wasserstein metric satisfies the triangle inequality[9], these could be used to assemble a distance matrix of triangle-relatedness where the smaller the values, the more similar the triangles.

c In turn, this was used to construct a dendrogram with optimal cuts determined using a Bayesian Hierarchical Clustering approach as described in Heller et al, ICML 2005[10]. The resulting cuts optimally grouped the 46 triangles into 7 distinct supra-triangle groups.

d-j Group members were optimally aligned before pooling and measuring separations as illustrated. Red ellipses represent 95% confidence intervals. Red spots are the median.

Supplementary Figure 6:

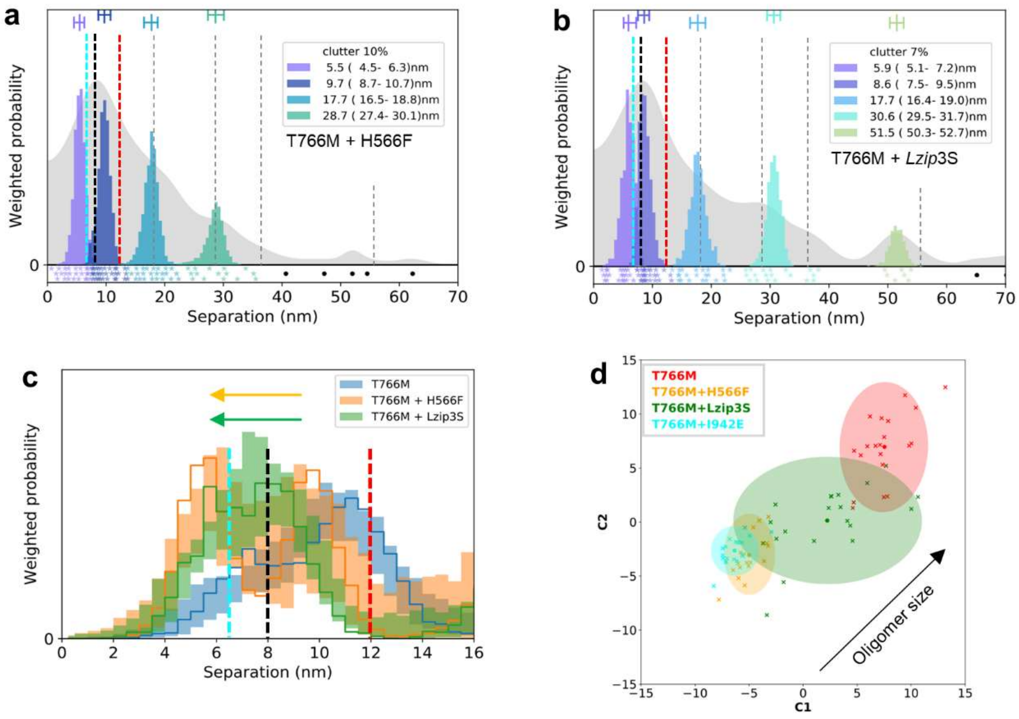

Supplementary Fig. 6. The H566F, and Lzip3S mutations interfere with T766M-EGFR oligomer formation, related to main text Fig. 7.

a, **b** FLImP analysis of 100 separation probability distributions between Affibody-CF640R pairs in the conditions indicated: Sum of posteriors of individual separations between fluorophores (gray background) and abundance-weighted probability distributions of individual components of decomposed separation distribution (colored peaks). Plot legend and bars above colored component distributions give the median and most-compact 68% confidence interval for each. Legend also gives median proportion of measurements assigned to clutter. The median peak positions marked by dashed lines are those of WT-EGFR.

c Comparisons between decomposed separation probability distributions between FLImP datasets. The continuous lines show the marginalized separation posterior, i.e., the sum of the abundance-weighted peaks, for each condition in the inset. The fluctuations around each continuous line arise from variations derived from FLImP decompositions for 20 bootstrap-resampled datasets to assess errors due to finite number of measurements. Horizontal colored arrows mark shifts in position of the peak, which are associated with the disruption in the $H2H^{ect}/2x^{kin}_{monomers}$ sub-unit induced by the mutations.

d Wasserstein MDS analysis of the FLImP decompositions for the conditions in the inset. The similarities or dissimilarities between the 21 separation sets associated with different conditions (one main FLImP decomposition plus 20 Bootstrapped with resampling decompositions) are compared. The axes in the plot are Component 1 (C1) and Component 2 (C2). C1 represents the dimension that captures the largest amount of variance in the data, while C2 represents the second-largest amount of variance that is orthogonal to C1. The center of the 95% confidence ellipses marks the positions of the main FLImP decompositions. The crosses mark the positions of individual bootstrapped separation sets.

Supplementary Figure. 7:

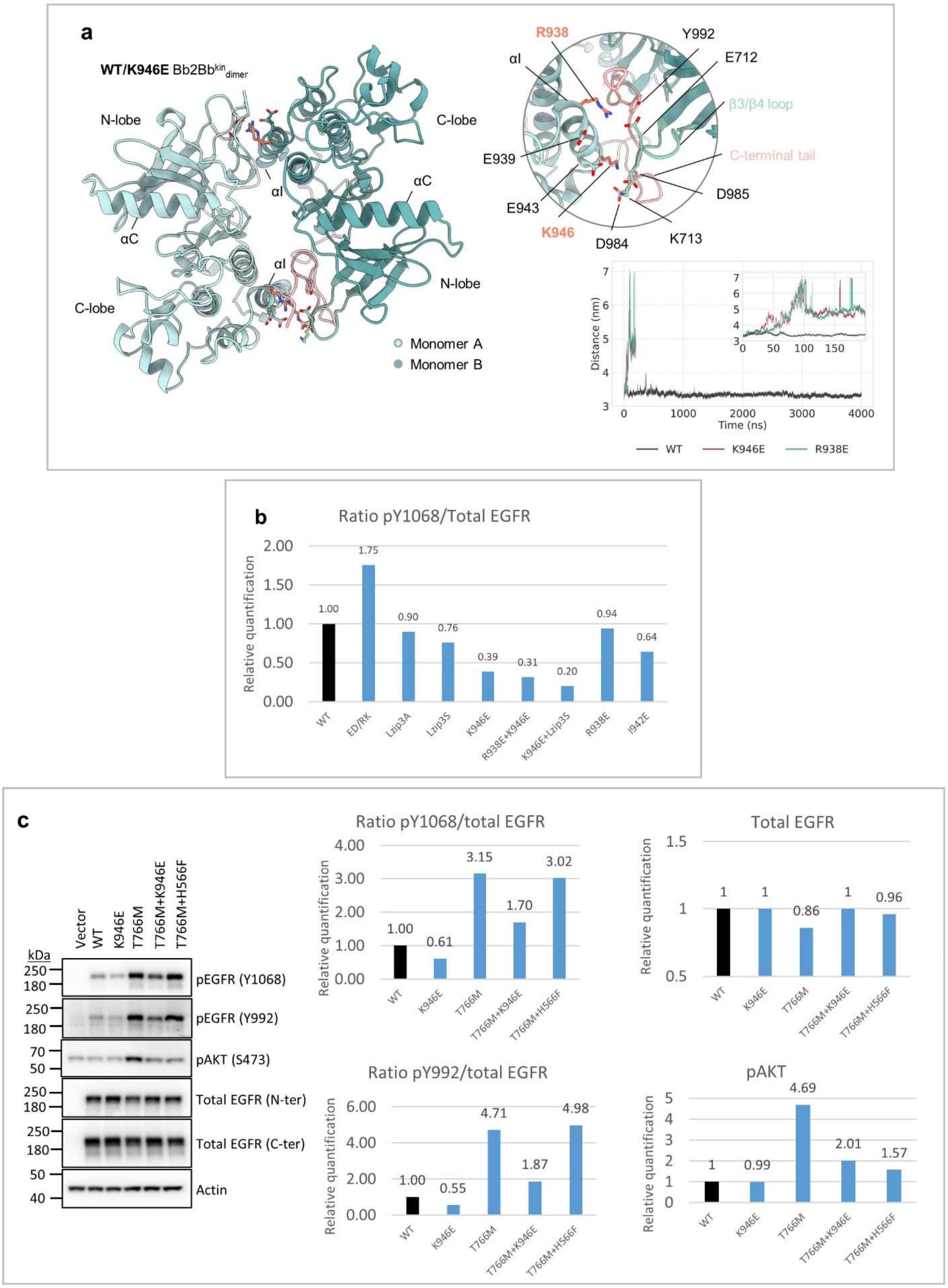

Supplementary Fig. 7. Modelling and simulations of the Bb2Bb$^{kin}_{dimer}$ sub-unit. Quantification Western Blot from main text Fig. 6d. Western Blot and its quantification of WT and T766M phosphorylation in the presence of the H566F and K946E mutations. Related to main text Fig. 8.

a Left, Cartoon representation of a Bb2Bb$^{kin}_{dimer}$ sub-unit (described in[11]) formed predominately through N-to-C lobe interactions (PDB ID: 3VJO [https://www.rcsb.org/structure/3VJO][12]). Top-Right, Close-up on the interface of the Bb2Bb$^{kin}_{dimer}$ sub-unit around the region where the K946E and R938E mutations lie (right). Both R938E and K946E are located on the αI-helix and are surrounded by residues that belong to flexible regions, namely the C-terminal tail and the β3/β4 loop. The presence of both positively and negatively charged residues in the vicinity of R938E and K946E already suggests that the introduction of a charge-reversal mutation within the two N-lobe/C-lobe interfaces is expected to have severe effects on the stability of the dimer. Bottom-right, Time series of the center-of-mass distance between monomers A and B over the course of the 4 µs long simulations of each variant, showing the detachment of the two monomers upon K946E or R938E mutation. Within the first 100 ns, the R938E and K946E dimers broke apart, unlike the WT, which remained intact for the entire 4 µs of simulation. In the case of K946E, although K713 of the β3/β4 loop interacts at the beginning with K946E and E943, as soon as K946E gets close enough to D984 and D985 of the C-terminal tail, the repulsive potential between the negatively charged side chains disrupts the connection of the monomers in one of the N-lobe/C-lobe interfaces, pushing the two monomers away. After that, it takes only a few ns for the monomers to become flexible enough to disrupt the connection on the second interface and eventually break apart. Interestingly, K946 itself does not seem to facilitate stable inter-monomer interactions as its sidechain mostly interacts with E943 of the αI-helix. Regarding the R938E mutant, the shorter and negatively charged sidechain of R938E prevents it from maintaining inter-monomer interactions seen between R938 and the sidechains of E712 and N996 in the WT.

b Quantification of the phosphorylation of the annotated mutant-EGFR in the absence of ligand in transfected CHO cells, which corresponds to the western blot in main text Fig. 8d.

c Western blot with quantification of the phosphorylation of the annotated mutant-EGFR expressed on Ba/F3 cells. Cells were grown in the absence of FBS and IL3 for 2 hours followed by western blotting to assess basal EGFR and AKT phosphorylation as a measure of their activity. The impairment of the T766M + H566F mutant to sustain Ba/F3 cell growth can be explained by its inability to activate a downstream signaling pathway in Ba/F3 cells. In the absence of IL3 and growth factors, we found that T766M + H566F expressing Ba/F3 cells were unable to phosphorylate and activate AKT signaling to the same extent as T766M. These blots shown are representative of n=3. Source data are provided as a source data file.

Supplementary Figure 8:

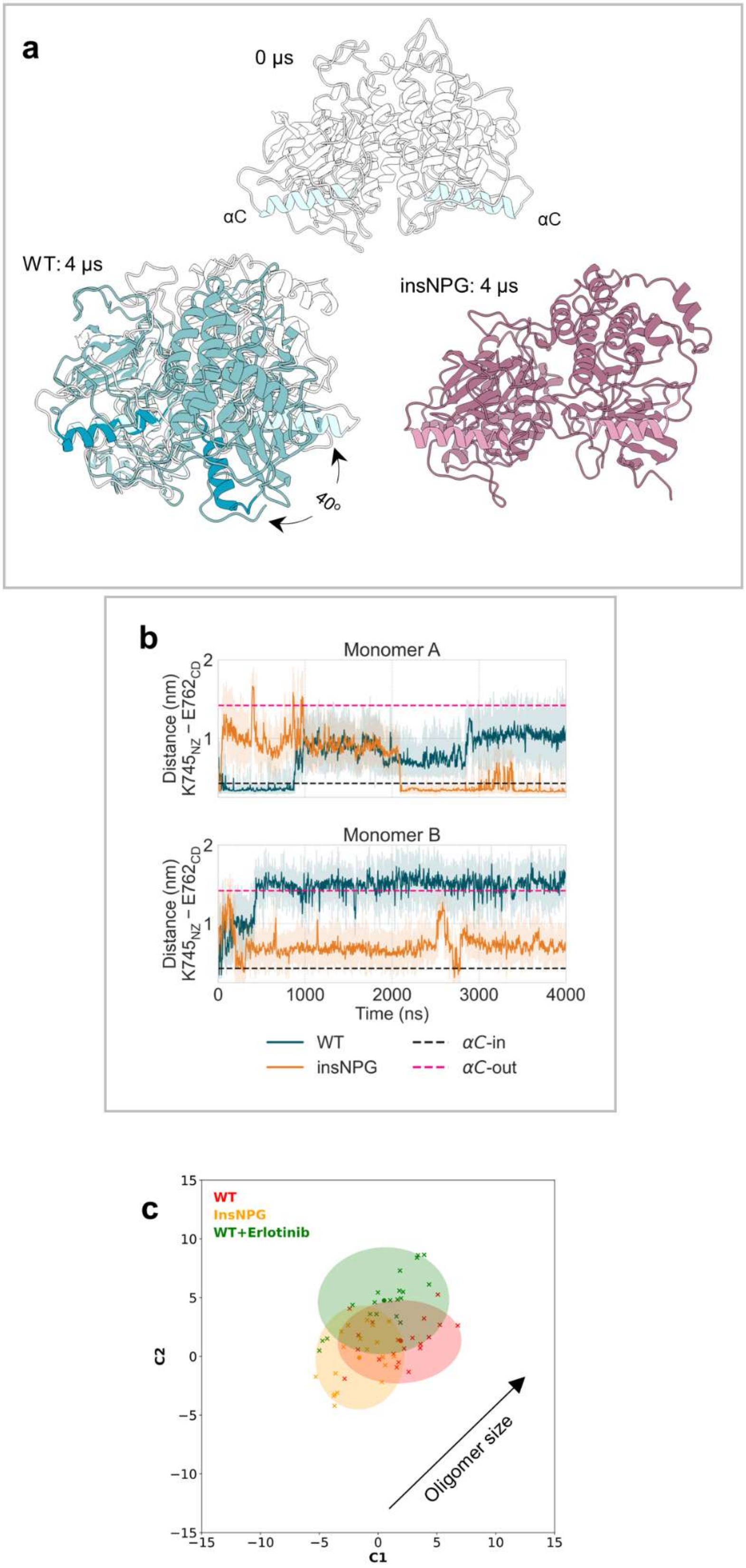

Supplementary Fig. 8. Modelling and simulations of the Bb2Bb$^{kin}_{dimer}$ sub-unit in Exon 20 mutations. Comparison between FLImP distributions for InsNPG and WT + Erlotinib. Related to main text Fig. 8.

a Snapshots at the beginning (0 µs) and the end (4 µs) of the simulations of a WT and InsNPG Bb2Bb$^{kin}_{dimer}$ sub-unit. The relative position of the two monomers of the InsNPG remains intact over the course of the simulation, compared to the WT, in which the one monomer rotates as a rigid body about 40° with respect to its initial position.

b Time series of the K745-E762 distance of each monomer of the Bb2Bb$^{kin}_{dimer}$ sub-unit. This distance was used as a proxy of the position of the αC-helix in the "αC-in" or "αC-out" conformation and shows the increased tendency of either monomer of the InsNPG (orange) to sample αC-in conformations, even in the absence of ATP. An indicative distance of the two residues in the αC-in (black) (PDB ID 2GS6 [https://www.rcsb.org/structure/2GS6][11]) and αC-out (pink) (PDB ID 2GS7 [https://www.rcsb.org/structure/2GS7][11]) conformation is shown with a dashed line as a reference distance.

c Wasserstein MDS analysis of the FLImP decompositions for the conditions in the inset. The similarities or dissimilarities between the 21 separation sets associated with different conditions (one main FLImP decomposition plus 20 Bootstrapped with resampling decompositions) are compared. The axes in the plot are Component 1 (C1) and Component 2 (C2). C1 represents the dimension that captures the largest amount of variance in the data, while C2 represents the second-largest amount of variance that is orthogonal to C1. The center of the 95% confidence ellipses marks the positions of the main FLImP decompositions. The crosses mark the positions of individual bootstrapped separation sets.

Supplementary Figure 9:

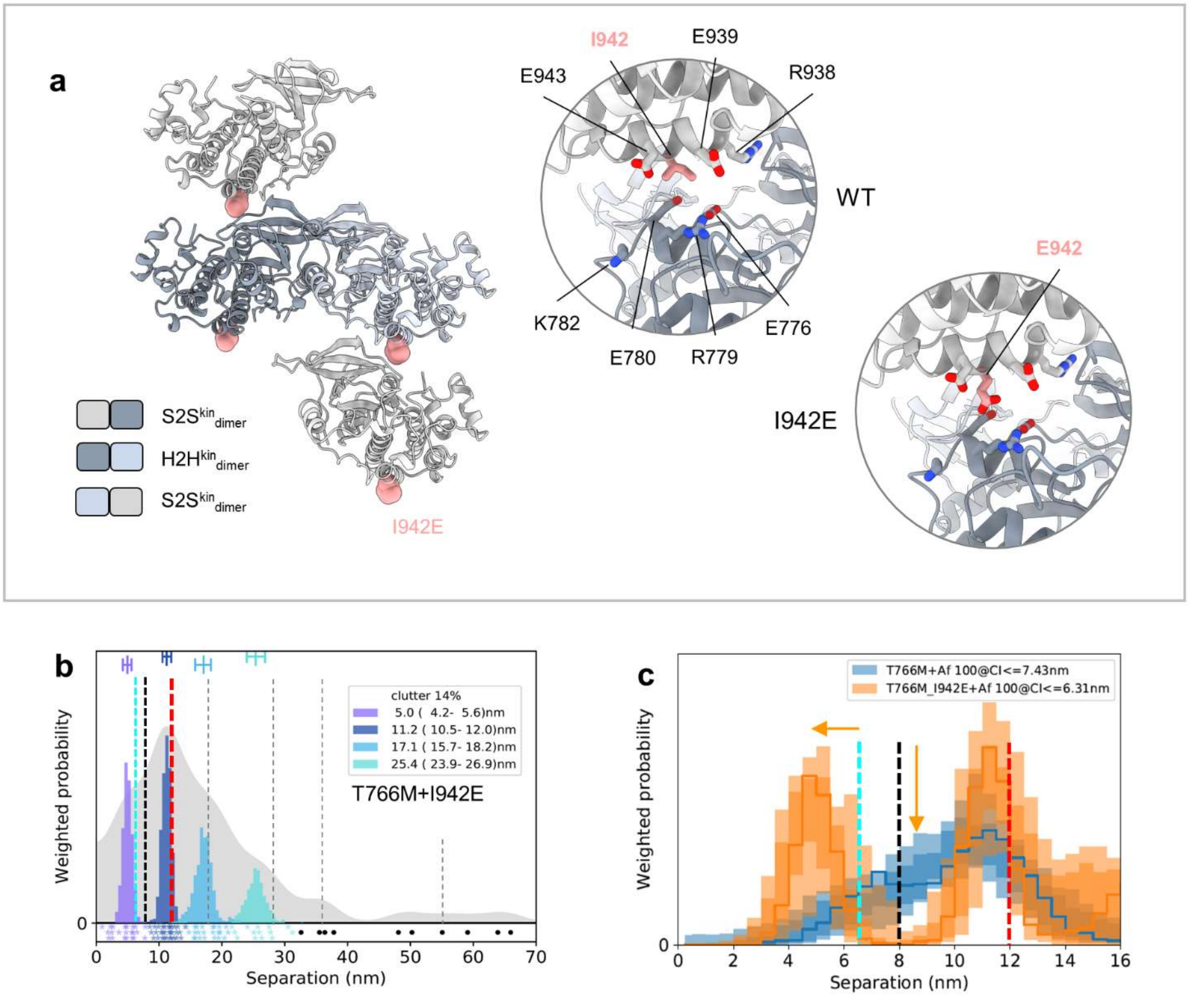

Supplementary Fig. 9. Modelling of a "zig-zag" tetramer and mutations that strengthen the S2S$^{kin}_{dimer}$, related to main text Fig. 9.

a Left, model of a "zig-zag" tetramer formed by two monomers forming two S2S$^{kin}_{dimer}$ sub-unit around an H2H$^{kin}_{dimer}$ sub-unit. In the crystal lattice of the kinase domain of the activator-impaired V948R EGFR (PDB ID 5CNO [https://www.rcsb.org/structure/5CNO][13]), two different dimers can be observed; one in which the interaction between the dimers is mediated primarily by interactions between the AP-2 helix in the C-terminal tail in one kinase and the N-lobe of the other (termed H2H$^{kin}_{dimer}$ sub-unit), and a second one where the β2-sheet and αD-helix of the one monomer interact with the αI- and αE-helices of the other monomer (termed S2S$^{kin}_{dimer}$ sub-unit). The decreased phosphorylation upon I942E mutation (main text Fig. 8d), which lies on the αI-helix of the one monomer and can form salt-bridges with R779 (αD-helix) and K782 (αG-αF loop) of the other monomer, highlights the biological relevance of the second dimer, which had been disregarded from the literature so far. The presence of these two kinds of dimers in the crystal lattice of the V948R EGFR made us speculate on the existence of a "zig-zag" kind of tetramer in cells composed of an H2H$^{kin}_{dimer}$ sub-unit and two monomers attached to it in an S2S$^{kin}_{dimer}$ way. What is more, in this type of tetramer, the two monomers that form the H2H$^{kin}_{dimer}$ are found in a Src-like inactive conformation, while the two monomers attached to the H2H$^{kin}_{dimer}$ sub-unit can adopt an active or inactive conformation, as their αC-helix and A-loop are not part of the S2S$^{kin}_{dimer}$ sub-unit interaction interface. Right, Close-up on the interface of the S2S$^{kin}_{dimer}$ sub-unit around the region where the I942E lies.

b FLImP analysis of 100 separation probability distributions between Affibody-CF640R pairs in the condition indicated: Sum of posteriors of individual separations between fluorophores (gray background) and abundance-weighted probability distributions of individual components of decomposed separation distribution (colored peaks). Plot legend and bars above colored component distributions give the median and most-compact 68% confidence interval for each. Legend also gives median proportion of measurements assigned to clutter. The median peak positions marked by dashed lines are those of WT-EGFR.

c Comparisons between decomposed separation probability distributions between datasets. The continuous lines show the marginalized separation posterior, i.e., the sum of the abundance-weighted peaks, for the conditions in the inset. The fluctuations around each continuous line arise from variations derived from FLImP decompositions for 20 bootstrap-resampled datasets to assess errors due to finite number of measurements. Horizontal arrow mark shifts in position associated with the $H2H^{ect}/2x^{kin}_{monomers}$ sub-unit induced by the mutations. Vertical arrow marks a reduction in density associated with the $St2St^{ect}/Asym^{kin}_{dimer}$ sub-unit.

Supplementary Fig. 10:

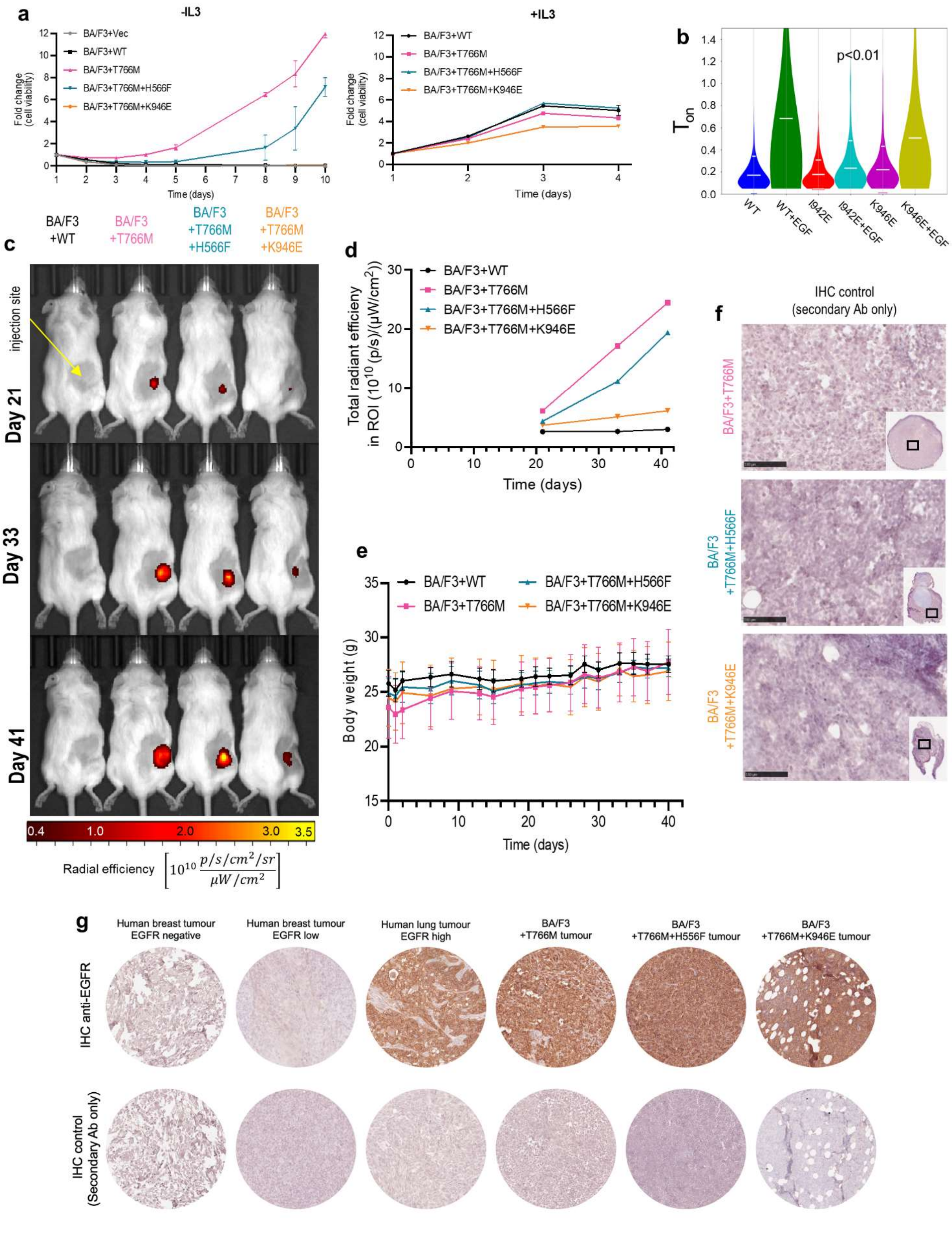

Supplementary Fig. 10. Growth controls in Ba/F3 cells and data supplementing the mice study, related to main text Fig. 10.

a Left, the cell viability over 10 days of Ba/F3 cell stable lines expressing WT-EGFR, T766M-EGFR, T766M + K946E-EGFR, T766M + H566F-EGFR or empty vector only, in the absence of IL3. Cells were deprived of IL3 5 days prior to cell counting and seeding. Measurements of ATP levels in lysed cells were taken on the day of seeding (day 1) and up to 10 days afterwards, using the Cell Titre Glo assay. Fold change reports the change in luminescence detected (mean +/- SD). IL3 independent proliferation is clearly only possible for the T766M-EGFR and T766M + H556F-EGFR expressing cell lines. The latter to a lesser extent. Ba/F3 cells expressing T776M + K946E-EGFR, WT-EGFR or empty vector only do not proliferate without IL3. Right, Positive controls showing more uniform growth among all conditions in the presence of IL3. N= 4 repeats.

b Two-color single particle tracking comparing T_{on} values. The data show that whilst the K946E mutation does not change the T_{on} value either of the ligand-free or ligand-bound state, the I942E significantly decreases the T_{ON} value of the ligand-bound state. Data acquired over at least 30 cells, over 3 independent biological replicates.

c One selected tumor-bearing animal per cohort was serially imaged by IVIS on days 21, 33, and 41 after tumor inoculation. Areas around injection sites were shaved, hence the different patterns in the fur of the animals.

d Quantitative epi-fluorescence data from the animals shown in **c** as a function of time.

e Cumulative body weights of animals in all cohorts throughout the experiment indicates expected growth and no obvious adverse effects that may have been reflected in body weight loss. There were also no significant differences in animals bearing different tumor types. N= 6 animals per cohort. Data are presented as mean values, and error bars = SD.

f Immunohistochemistry control staining of tumor sections from the same tumors as in main text Fig. 10 with all reagents except for the specific anti-EGFR antibody. Micrographs show background staining. Scale bars are 100 µm.

g Top, Immunohistochemistry staining of tumor sections from a low EGFR expressing breast cell line and a high EGFR expressing NCI-H1975 lung cancer cell line (EGFR levels were independently confirmed by pathologists' annotation) compared with tumor sections from the Ba/F3 cells expressing T766M, T766M+H566F, and T766M+K946E EGFR mutants, all using the same specific anti-EGFR antibody as in main text Fig. 10. To estimate the number of receptors in the Ba/F3 tumors, we noted that the relative number of EGFR gene copies in H1975 cells measured by the nanofluidic digital PCR array is 1.8x that of A549[14]. In A549 cells, surface plasmon resonance data found a density of cell surface receptors of 142 receptors/μm^2, corresponding to 0.11 million per cell[15]. Thus, according to both data sets, the copy numbers of EGFR in H1975 cells is 1.8 x 0.11 = 198,000 receptor copies/cell. Our results indicate a higher expression in the Ba/F3 tumors than in the H1975 ones. Bottom, control staining of sections from the same tumors with all reagents except for the specific anti-EGFR antibody. <u>Source data are provided as a source data file.</u>

Supplementary Table 1. Reliability and reproducibility checklist for molecular dynamics simulations

Questions:	Yes	N/A	Comments:
**1. Convergence of simulations and analysis**			
1a. Is an evaluation presented in the text to show that the property being measured has equilibrated in the simulations (*e.g.* time-course analysis)?	☒		We did not include one in the main text, but we provide one here below for the case of the ectodomain simulations (**Time series of the Cα root mean square deviation (RMSD) of the H2H and B2B ectodomain simulations**). Given the dissociation observed in the kinase domain simulations of R938E and K946E, such an analysis would not be meaningful as the systems get out of equilibrium and eventually adopt a conformation that results from monomers crossing the periodic boundaries.
1b. Then, is it described in the text how simulations are split into equilibration and production runs and how much data were analyzed from production runs?	☒		Details on the length of the equilibration and production steps are given in the "Supplementary Note 1. MD simulations and structural modelling" section of the Supplementary Information.
1c. Are there at least 3 simulations per simulation condition with statistical analysis?		☒	Given the size of the studied systems and the resulting high computational cost, we were unable to have 3 independent simulations per studied system. The presented results come from a single replica.
1d. Is evidence provided in the text that the simulation results presented are independent of initial configuration?		☒	Given the size of the studied systems and the resulting high computational cost, we were unable to have independent simulations starting from different initial configurations. The simulations were initiated from configurations that were modelled based on experimentally derived configurations to have the best possible biological relevance of the sampled conformational space.
**2. Connection to experiments**			
2a. Are calculations provided that can connect to experiments (*e.g.* loss or gain in function from mutagenesis, binding assays, NMR chemical shifts, J-couplings, SAXS curves, interaction distances or FRET distances, structure factors, diffusion coefficients, bulk modulus and other mechanical properties, *etc.*)?	☒		In Supplementary Figure 2c, we report the distance between the centre of the mass distance of the DIII domains where the fluorescent affibody is expected to bind, which is then compared with the FLImP fingerprints of WT EGFR in Figure 2a and discussed in the "$H2H^{ect}_{dimer}/2x^{kin}_{monomers}$ are sub-units in $hetero^{conf}$-oligomers" section of the main text.
**3. Method choice**			
3a. Is it described in the text what force field and water model are used and why?	☒		Details about the protein/membrane force field (CHARMM36m) and water model (TIP3P-CHARMM) used in each simulation can be found in Supplementary Note 1. MD simulations and structural modelling. The choice of this force field resulted from our decision to simulate glycosylated models, which led us to use the CHARMM force

			field family for protein, membrane, water, and glycans as implemented in the CHARMM-GUI server that we used to build the simulated systems. There is a big body of work with glycosylated membrane proteins (see in COVID-19 literature for e.g.[16]) that have used this combination of force fields and have shown that it can reproduce experimentally observed properties.
3b. Do simulations contain membranes, membrane proteins, intrinsically disordered proteins, glycans, nucleic acids, polymers, or cryptic ligand binding?	☒		They contain both a POPC/cholesterol/ n-palmitoyl-sphingomyelin (30:11:2 ratio) membrane and glycans. The glycan composition and glycosylated sites are described in the "Ectodomain mutations – H2H^{ect} simulations" section of the Supplementary Information.
If 3b is **YES**, are enhanced sampling methods used?		☒	The size and complexity of the systems would make the application of enhanced sampling techniques difficult to converge. Moreover, obtaining a converged landscape was out of the scope of this study, given the simulations were used to help with the assignment of the FLImP profiles to structural ensembles, without studying the transition between them.
**4. Code and reproducibility**			
4a. Is a table provided describing the system setup, such as simulation box dimensions, total number of atoms, total number of water molecules, salt concentration, lipid composition (number of molecules and type)?	☒		Refer to. Supplementary Table 2
4b. Is it described in the text what simulation and analysis software and which versions are used?	☒		All simulations were run using the GROMACS v.2020.3 package. The analysis of the simulations was done using the MDTraj 1.9.4 Python library.
4c. Are initial coordinate and simulation input files and a coordinate file of the final output provided as supplementary files or in a public repository?	☒		We have deposited all the necessary files (structure file, topology files, simulation parameters) to reproduce the results in the following YARETA repository:<br>DOI:<br>10.26037/yareta:qtkuoibmhndc3jxcwtwzo7eeey;<br>ID: 2f052659-2a39-4d10-bb43-b6dea49ed2d9[17]
4d. Is there custom code or custom force field parameters?		☒	We used a standard CHARMM36m force field for the protein and lipids, as implemented in the CHARMMGUI server.
If **YES**, are they provided as supplementary profiles or in a public repository?		☒	-

Time series of the Cα root mean square deviation (RMSD) of the H2H and B2B ectodomain simulations.

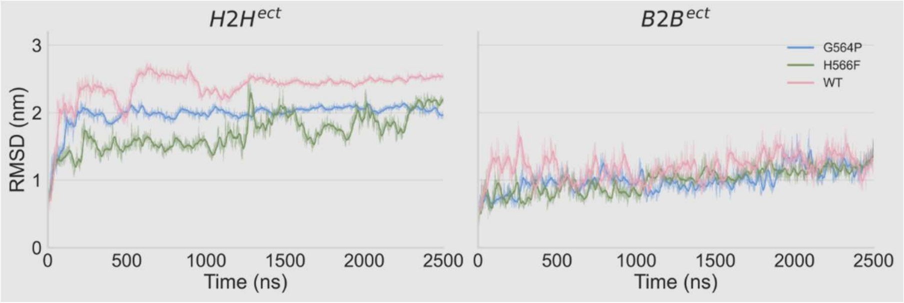

Supplementary Table 2: System details of the simulated systems. The exact files can also be found in the repository where we have deposited all the input files for the MD simulations (YARETA, DOI: 10.26037/yareta:qtkuoibmhndc3jxcwtwzo7eeey[17]).

	Simulated system	Simulation box dimensions (nm)[a]	Total num. of atoms	Total num. of water molecules	NaCl conc. (M)	Lipid composition, num. of molecules (POPC/CHOL/PSM)[b,c]
H2H^{ect}	WT	22.7 x 22.7 x 24.9	1,326,126	352,632	0.15	1440/528/96
	G564P	22.4 x 22.3 x 21.0	1,085,233	272,713	0.15	1440/528/96
	H566F	22.5 x 22.5 x 20.7	1,085,241	272,707	0.15	1440/528/96
B2B^{ect}	WT	23.1 x 23.1 x 26.9	1,482,317	404,269	0.15	1440/528/96
	G564P	25.0 x 25.0 x 24.7	1,473,945	401,477	0.15	1440/528/96
	H566F	23.3 x 23.3 x 26.4	1,473,356	401,286	0.15	1440/528/96
Bb2Bb$^{kin}_{dimer}$	WT	11.4 x 10.7 x 9.3 x 0.0 x 0.0 x 3.8 x 0.0 x -3.8 5.4	117,793	35,749	0.15	-
	R938E	11.4 x 10.7 x 9.3 x 0.0 x 0.0 x 3.8 x 0.0 -3.8 x 5.4	117782	35,750	0.15	-
	K946E	11.4 x 10.7 x 9.3 x 0.0 x 0.0 x 3.8 x 0.0 -3.8 x 5.3	117801	35,755	0.15	-
	D770-N771insNPG	11.4 x 10.7 x 9.3 x 0.0 x 0.0 x 3.8 x 0.0 -3.8 x 5.3	115619	35,001	0.15	-

[a] The ectodomain systems were simulated in a cubic box, while the kinase domain in an octahedral one. [b]POPC: 1-palmitoyl-2-oleoyl-sn-glycero-3-phosphocholine, CHOL: cholesterol, PSM: n-palmitoyl-sphingomyelin.

[c] The lipids were distributed symmetrically to each lipid bilayer. The reported number of lipids corresponds to the total lipids per bilayer and not per leaflet.

Supplementary Table 3:

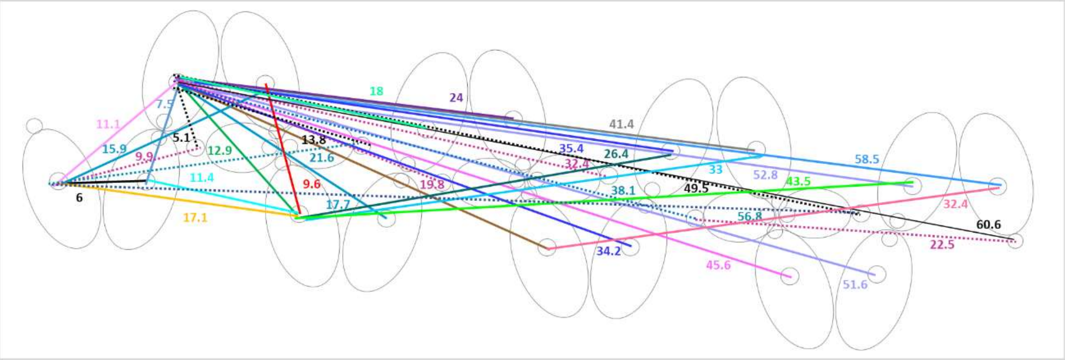

Dimer conformer sub-units	H2H^ect	H2H^ect	H2H^ect	S2S^ect	B2B^ect	B2B^ect	B2B^ect	B2B^ect	◄ Oligomer separations ►																			
Predicted separations from model	4-4.5	4.8-5.4	5.7-6.3	7.2-7.8	9.3-10.2* (× 2)	10.8-11.7* (× 2)	12.6-13.2	13.5-14.1	15.6-16.2	16.9-18.3* (× 3)	19.5-20.1	21.3-21.9	22.2-22.8	23.7-24.3	26.1-26.7	28.5-29.2	32.1-33.3* (× 2)	33.9-34.5	35.1-35.7	37.8-38.4	41.1-41.7	45.3-45.8	49.2-49.8	51.3-51.9	52.5-53.1	56.5-57.1	58.2-58.8	60.3-60.9
WT			✓	✓		✓	✓			✓						✓			✓							✓		
ED/RK		✓	✓	✓					✓								✓											
T766M			✓	✓	✓	✓	✓				✓					✓						✓				✓		
WT+mAb2E9		✓	✓	✓	✓	✓	✓	✓	✓			✓	✓				✓						✓					
WT+mAb108			✓		✓	✓	✓	✓			✓				✓	✓												
WT+Erlotinib			✓	✓	✓	✓	✓	✓	✓	✓	✓	✓	✓	✓	✓		✓	✓	✓	✓						✓	✓	
ΔC			✓	✓			✓	✓		✓	✓				✓	✓					✓		✓	✓				
H566F	✓	✓	✓	✓		✓	✓	✓	✓	✓						✓	✓											
WT+Lapatinib	✓	✓		✓	✓	✓	✓	✓	✓	✓				✓			✓	✓	✓								✓	✓
*Lzip*3S	✓	✓	✓	✓	✓	✓							✓	✓					✓	✓								
ED/RK+L680N	✓			✓			✓	✓					✓	✓														
ED/RK+L680N+*Lzip*3S			✓			✓	✓	✓				✓	✓						✓	✓			✓	✓				
K946E	✓		✓	✓		✓	✓	✓						✓	✓													
T766M+K946E	✓	✓	✓	✓	✓			✓	✓						✓	✓	✓						✓					
insNPG	✓	✓	✓	✓	✓	✓				✓	✓		✓	✓			✓	✓	✓					✓	✓			
I942E	✓	✓			✓	✓		✓	✓			✓							✓									
*Lzip*3S+K946E		✓	✓	✓	✓	✓	✓	✓	✓						✓	✓				✓	✓							✓
*Lzip*3S+L680N	✓	✓			✓	✓			✓	✓						✓								✓	✓			
G564P	✓	✓	✓	✓	✓	✓					✓	✓	✓				✓	✓										
G564P+ED/RK	✓	✓		✓			✓	✓	✓						✓					✓	✓		✓	✓	✓	✓	✓	
*Lzip*3A		✓	✓			✓	✓	✓	✓	✓						✓					✓	✓						✓
T766M+H566F	✓	✓			✓					✓						✓												
T766M+*Lzip*3S**		✓	✓	✓						✓																		
T766M+I942E	✓	✓				✓			✓	✓				✓	✓													

Supplementary Table 3. Separations predicted by the model and those satisfied by mutations and treatments, highlighting changes from cancer mutations. Top row**,** Separations predicted by the model shown above the table. These separations are calculated from the 6 nm separation between labeled sites in the WT-H2H$^{ect}_{dimer}$ [1]. We associated an error of ± 0.3 nm (1 mm) to the measurements. (*****) Separation range that encompasses two (x2) or three (x3) unresolved separations in the model based the <3 nm resolution of our measurements. Column 1, Mutations examined by FLImP. In black, conditions that satisfy the zig-zag model above the table. In green conditions expected to satisfy a repeat of H2H$^{ect}_{dimers}$ forming a homo-oligomer. Predicted separations identified in each data set are marked by Ticks. (******) one separation decomposed in the set from T766M+Lzip3S could not be associated to a predicted separation, being shorter by 0.3 nm. All other separations were accommodated (ticks). Columns 2 to 9, separations either arising or directly dependent on one of the three ligand-free dimer conformers, as annotated in top row. Second column, Ticks appear in conditions observed to induce a substantial conformational change in the H2H$^{ect}_{dimer}$ (see main text). Pink background, Dimer associated separations that increase in prevalence in the corresponding EGFR mutations compared with wild type EGFR. Aqua background, Dimer associated separations that increase in prevalence in the corresponding T766M+ another EGFR mutation, compared with T766M-EGFR. Grey background, Separations not found in the condition. This can be explained by the sampling nature of the method and the large number of possible separations.

Supplementary Table 4. Primers used in Site-Directed Mutagenesis experiments or to PCR amplify EGFR

Primer
Primer for L680N site-directed mutagenesis:<br>Forward: 5’- GAA GCT CCC AAC CAA GCT CTC AAT AGG ATC TTG -3’<br>Reverse: 5’ – TTC AGT TTC CTT CAA GAT CCT ATT GAG AGC TTG – 3’
Primer for G564P site-directed mutagenesis:<br>Forward: 5'-TGA CGC AGT GGG GGG GGTCAA TGT AGT GGG-3'<br>Reverse: 5'-CCC ACT ACA TTG ACC CCC CCC ACT GCG TCA-3'
Primer for K946E site-directed mutagenesis:<br>Forward: 5'-GGG GGT CTC GGG CCA TCT CGG AGA ATT CGA TGA TC-3'<br>Reverse: 5'-GAT CAT CGA ATT CTC CGA GAT GGC CCG AGA CCC CC-3'
Primer for R938E site-directed mutagenesis:<br>Forward: 5'-GGA GAA TTC GAT GAT CAA CTC CTC GAA CTT TGG GCG ACT ATC TGC-3'<br>Reverse: 5'-GCA GAT AGT CGC CCA AAG TTCGAG GAG TTG ATC ATC GAA TTC TCC-3'
Primer for Lzipx3A site-directed mutagenesis:<br>Forward: 5'-CGC ATG AAG GCG CCG ATC CCC GCG GCC ACC GCC AGC AGC A-3'<br>Reverse: 5'-TGC TGC TGG CGG TGG CCG CGG GGA TCG GCG CCT TCA TGC G-3'
Primer for Lzipx3S site-directed mutagenesis:<br>Forward: 5'-CCT TCG CAT GAA GCT GCC GAT CCC CGA GGC CAC CGA CAG CAG CAA GAG G-3'<br>Reverse: 5'-CCT CTT GCT GCT GTC GGT GGC CTC GGG GAT CGG CAG CAG CTT CAT GCG AAG G-3'
Primer for E10005R + D10006K (ED/RK) site-directed mutagenesis:<br>Forward: 5'-CAC CAC GTC GTC CAT CTT TCT TTC ATC CAT CAG GGC ACG GTA GAA GTT G-3'<br>Reverse: 5'-CAA CTT CTA CCG TGC CCT GAT GGA TGA AAG AAA GAT GGA CGA CGT GGT G-3'
Primer for T766M site-directed mutagenesis:<br>Forward: 5'-CCT CCA CCG TGC AGC TCA TCA TGC AGC TCA TGC CCT TCG GC-3'<br>Reverse: 5'-GCC GAA GGG CAT GAG CTG CAT GAT GAG CTG CAC GGT GGA GG-3'
Primer for I942E site-directed mutagenesis:<br>Forward: 5'-TCG GGC CAT TTT GGA GAA TTC CTC GAT CAA CTC ACG GAA CTT TGG-3'<br>Reverse: 5'-CCA AAG TTC CGT GAG TTG ATC GAG GAA TTC TCC AAA ATG GCC CCGA-3'
Primer for H566F site-directed mutagenesis:<br>Forward: 5'-GCA GGT CTT GAC GCA GAA GGG GCC GTC AAT GTA G-3'<br>Reverse: 5'-CTA CAT TGA CGG CCC CTT CTG CGT CAA GAC CTG C-3'
Primer for InsNPG site-directed mutagenesis:<br>Forward: 5’-GGC TAG CGT GGA CAA CCC CGG CAA TCC TCA CGT GTG CCG CC-3'<br>Reverse: 5’- GGC GGC ACA CGT GAG GAT TGC CGG GGT TGT CCA CGC TAG CC-3'
EGFR PCR:<br>Forward: 5’- TAA GCA GCT AGC ACC ACC ATG CGA CCC TCC GGG ACG GC -3'<br>Reverse: 5’- TAA GCA GCG GCC GCT CAT GCT CCA ATA AAT TCA C -3'

Supplementary Table 5. Parameters of the 1-way ANOVA analysis with Tukey's multiple comparison correction performed. Number of comparisons per family = 6, α=0.05, related to main text Fig. 10.

TUMOR FLUORESCENCE BY IVIS (Fig. 10c)

	Mean Diff	95,00% CI of diff	Significant	Summary	P Value
WT vs. T766M	-39.63	-60.13 to -19.12	Yes	***	0.0001
WT vs. T766M+H566F	-34.99	-55.49 to -14.48	Yes	***	0.0006
WT vs. T766M+K946E	-9.607	-30.11 to 10.90	No	ns	0.5667
T766M vs. T766M+H566F	4.642	-15.86 to 25.15	No	ns	0.92
T766M vs. T766M+K946E	30.02	9.517 to 50.53	Yes	**	0.0029
T766M+H566F vs. T766M+K946E	25.38	4.876 to 45.89	Yes	*	0.012

	Mean 1	Mean 2	Mean Diff	SE of diff	n1	n2	q	DF
WT vs. T766M	5.082	44.71	-39.63	7.326	6	6	7.65	20
WT vs. T766M+H566F	5.082	40.07	-34.99	7.326	6	6	6.754	20
WT vs. T766M+K946E	5.082	14.69	-9.607	7.326	6	6	1.854	20
T766M vs. T766M+H566F	44.71	40.07	4.642	7.326	6	6	0.896	20
T766M vs. T766M+K946E	44.71	14.69	30.02	7.326	6	6	5.796	20
T766M+H566F vs. T766M+K946E	40.07	14.69	25.38	7.326	6	6	4.899	20

TUMOR WEIGHT (Fig. 10e)

	Mean Diff	95,00% CI of diff	Significant	Summary	P Value
WT vs. T766M	-0.5439	-0,7511 to -0.3366	Yes	****	<0.0001
WT vs. T766M+H566F	-0.2139	-0.4211 to -0.006597	Yes	*	0.0416
WT vs. T766M+K946E	-0.01822	-0.2255 to 0.1890	No	ns	0.9946
T766M vs. T766M+H566F	0.33	0.1228 to 0.5373	Yes	**	0.0013
T766M vs. T766M+K946E	0.5257	0.3184 to 0.7329	Yes	****	<0.0001
T766M+H566F vs. T766M+K946E	0.1956	-0.01162 to 0.4029	No	ns	0.0686

	Mean 1	Mean 2	Mean Diff,	SE of diff,	n1	n2	q	DF
WT vs. T766M	0.00055	0.5444	-0.5439	0.07405	6	6	10.39	20
WT vs. T766M+H566F	0.00055	0.2144	-0.2139	0.07405	6	6	4.084	20
WT vs. T766M+K946E	0.00055	0.01877	-0.01822	0.07405	6	6	0.3479	20
T766M vs. T766M+H566F	0.5444	0.2144	0.33	0.07405	6	6	6.303	20
T766M vs. T766M+K946E	0.5444	0.01877	0.5257	0.07405	6	6	10.04	20
T766M+H566F vs. T766M+K946E	0.2144	0.01877	0.1956	0.07405	6	6	3.736	20

Supplementary Note 1. MD simulations and structural modelling

The initial coordinate and simulation input files to reproduce the simulations described below have been deposited in the following YARETA repository[17]:

DOI: 10.26037/yareta:qtkuoibmhndc3jxcwtwzo7eeey

Ectodomain mutations – H2H^{ect} simulations

To simulate the H2H$^{ect}_{dimer}$ sub-unit, we used the model of the asymmetric dimer seen in the crystal packing of the PDB entry 4KRP [https://www.rcsb.org/structure/4KRP][18] after removing 9G8-NB and adding the TM helix that we used in our previous work.[1] G564P and H566F mutations in each monomer were introduced to the WT structure using MODELLER.[19] The WT and mutant models were N-glycosylated at N151, N172, N328, N337, N389, N420, N504, N544, N579 with the core glycan (Man3GlcNAc2-Asn) and one site (N32) with the "Pauci-mannose" glycan type (Man3GlcNAc2(Fuc)-Asn) as this site has been reported to be fucosylated.[20] *In-silico* glycosylation was carried out using the CHARMM-GUI Glycan Modeler tool and the CHARMM carbohydrate force field was used in the performed simulations.[21,22]

To create a realistic membrane environment, we embedded the glycosylated ECD in a lipid bilayer mimicking the lipid composition found in CHO cells, i.e. with a ratio of 1-palmitoyl-2-oleoyl-sn-glycero-3-phosphocholine (POPC), cholesterol (CHOL), and n-palmitoyl-sphingomyelin (PSM) of 30 : 11 : 2. [23] A symmetric 250 Å x 250 Å lipid bilayer patch was generated using CHARMM-GUI's input generator.[24,25] The generated model was parameterized using the CHARMM36m force field [26] at pH 7.4 and CHARMM36 parameters were used for the lipids and glycans as provided by CHARMM-GUI. To ensure consistency, the protonation states of the residues remained the same across all systems. The generated model was parameterized using the CHARMM36m force field[26] at pH 7.4 and CHARMM36 parameters were used for the lipids and glycans as provided by CHARMM-GUI. The systems were solvated with modified TIP3P-CHARMM water molecules,[27] and Na^+ and Cl^- ions were added to reach neutrality and a final ion concentration of 0.15 M.

All simulations were run using the GROMACS v.2020.3 package.[28] Initial energy minimization was performed using the steepest-descent algorithm, followed by a six-step equilibration protocol in the NVT and NPT ensembles provided by CHARMM-GUI. Lipid, glycan, and protein atom restraints were gradually relaxed during equilibration, with temperature and pressure maintained at 310 K and 1 bar, respectively, using velocity-rescale thermostat[29] and Parrinello-Rahman barostat[30]. Bond lengths to hydrogen atoms were constrained by the LINCS algorithm,[31] and van der Waals interactions had a cutoff of 12 Å. Electrostatic interactions were computed using the particle mesh Ewald method[32] with a cutoff of 12 Å and Fourier spacing of 1.6 Å. Following a constrained equilibration of 5 ns in the NVT ensemble and 10 ns in the NPT ensemble, production runs for each system (WT and mutants) were conducted for a duration of 2.5 µs without applying any restraints.

Ectodomain mutations – B2B^{ect} simulations

To simulate the B2B$^{ect}_{dimer}$ sub-unit, we used the crystal structure of the dimer from the PDB entry 3NJP [https://www.rcsb.org/structure/3NJP][33]. We coupled the ectodomain to the TM helices in the Nter+ conformation, as described in the PDB entry 2M20 [https://www.rcsb.org/structure/2M20][34], using MODELLER.[16] G564P and H566F mutations in each monomer were introduced into the WT structure using MODELLER.[16] For consistency with the H2H^{ect} simulations, the WT and mutant models were N-glycosylated at the same sites as described above, and the systems were embedded in a POPC/CHOL/PSM membrane – as in the H2H^{ect} simulations. The same parameterization (choice of force field, salt concentration, etc.) and simulation protocols (equilibration and production steps) were followed as in the H2H^{ect} simulations The production runs were carried out for 2.5 µs for each system.

Kinase mutations – Bb2Bb$^{kin}_{dimer}$ sub-unit simulations

EGFR kinase simulations starting from the Bb2Bb$^{kin}_{dimer}$ sub-unit were based on the dimers seen in the crystal packing in PDB entry 3VJO [https://www.rcsb.org/structure/3VJO].[12] The co-crystalized ligand was removed from both monomers and missing atoms were built with MODELLER[19]. Each dimer consisted of the sequence G696-A1022 (in the numbering with the 24-aa tag). The two non-naturally occurring R938E and K946E mutations, as well as the D770-N771InsNPG (InsNPG) mutation on the αC/β4 loop, were introduced in each monomer of the WT dimer structure with MODELLER[19]. In the case of the InsNPG, the structure of each monomer of the resulting modelled dimer was then compared to the crystal structure of InsNPG in complex with a covalent inhibitor (PDB ID 4LRM [https://www.rcsb.org/structure/4LRM]) to ensure that the overall conformation of each monomer and the environment around the point of the mutation are in accordance with the experimentally derived structure.

For the unbiased MD simulations, each simulated system was parameterized using the CHARMM36m force field[26] at pH 7.4. The protonation states of the residues were determined by PlayMolecule[36], which maintained the usual charge states for all molecules. The systems were solvated with modified TIP3P-CHARMM water molecules[26] in a dodecahedral box, while Na^+ and Cl^- ions were added to reach a final concentration of 0.15 M.

GROMACS v.2020.3 package[28] was used for all simulations. Prior to production simulations, the energy of each system was minimized through steepest-descent energy minimization. After minimization, the initial velocities for the atoms were taken from Maxwell distribution at 300 K, and the system was simulated for 5 ns at the NVT ensemble using a velocity rescaling thermostat[29] and position restraints on heavy atoms (1000 kJ mol^{-1} nm^{-2}), followed by 10 ns in the NPT ensemble under constant pressure (1 bar) using the Berendsen barostat[37], followed by 5 ns using the Parrinello-Rahman barostat.[30] All bond lengths to hydrogen atoms were constrained using the LINCS algorithm,[31] while van der Waals interactions were treated with a cut-off of 12 Å. Electrostatic interactions were computed using the particle mesh Ewald method[32] with a direct sum cut-off of 12 Å and the Fourier spacing of 1.6 Å. Each production run was 4 µs long.

S2S$^{kin}_{dimer}$ sub-unit modelling

The crystal lattice of the activator-impaired V948R EGFR (PDB ID 5CNO [https://www.rcsb.org/structure/5CNO]) reveals two distinct dimers: an H2H$^{kin}_{dimer}$ sub-unit and an S2S$^{kin}_{dimer}$ sub-unit.[13] The decreased phosphorylation upon I942E mutation (main text Fig. 8d), which lies on the αI-helix of the one monomer and can form salt-bridges with R779 (αD-helix) and K782 (αG-αF loop) of the other monomer, highlights the biological relevance of the second dimer sub-unit, which had been disregarded from the literature so far. The presence of these two kinds of dimers in the crystal lattice of the V948R EGFR prompt speculation about the existence of a "zig-zag" tetramer in cellular contexts, comprising of an H2H$^{kin}_{dimer}$ sub-unit and two monomers attached to it in a S2S$^{kin}_{dimer}$ sub-unit arrangement (main text Fig. 9c, Supplementary Fig. 9). In this proposed tetramer model, the two monomers of the H2H$^{kin}_{dimer}$ sub-unit are found in an Src-like inactive conformation, while the two monomers attached to the H2H$^{kin}_{dimer}$ sub-unit can adopt an active or inactive conformation, as their αC-helix and A-loop are not part of the S2S$^{kin}_{dimer}$ sub-unit interaction interface. This flexibility suggests that S2S$^{kin}_{dimer}$ sub-unit can be formed and attached to a central Asym$^{kin}_{dimer}$ sub-unit. This aligns with our proposed model of ligand-free hetero^{conf}-oligomerization (main text **Fig. 9b**), where inactive dimers can act as spacers between active asymmetric kinase dimers.

Supplementary Note 2. FLImP automated data acquisition

When imaging cells of interest, the following procedure was followed:

Focusing - an additional focal polishing step was applied for prolonged data acquisitions. This involved recording a z-stack of images over +/- 2 µm with 200 nm step, extracting diffraction-limited objects using a 3x3 pixel top-hat filter, cross-correlating each frame with a 2D Gaussian kernel approximating the

experimentally determined PSF size, and finding the minimum of a second-order polynomial to estimate the focal plane.

ROI detection and imaging - ~6000 fields of view (FOV) were evaluated for FLImP suitability in each well at 0.1 mm intervals. FOV suitability was determined using a classical image segmentation approach whereby the presence of cells in the field was determined by the extent of Hoechst or GFP labeling, evaluated using Otsu thresholding followed by removal of objects too small to be nuclei/cells. The resulting binary mask was used to calculate the fraction of Hoechst or GFP labeling in the FOV. If this fraction exceeded a specific threshold, the extent of FLImP labeling within cells was evaluated using the Triangle method of thresholding. Resulting binary mask was multiplied by the cell-area binary mask to produce a measure of FLImP-labelling extent. If the fraction of FLImP labeling exceeded a pre-defined threshold, a FLImP series was recorded for the current FOV. Otherwise, the system moved to the next FOV. Single images of FOVs were recorded for post-acquisition user validation and the evaluation process typically took no more than 2 seconds per FOV. Finally, for suitable FOVs, a FLImP series acquisition was made comprising 1200 frames with 20 ms exposure time and used 20 mW 640 nm laser power.

Supplementary Note 3. FLImP data analysis

Section I. FLIMP ANALYSIS PROCESS OVERVIEW

The FLImP analysis process is divided in the stages very briefly outlined here, and then described in detail in the next section. Note that the new methods described here and in Supplementary Note2 are a set of algorithms to reimplement and refine in an automated fashion our established processes[1,2,38–41] to acquire, identify and analyze tracks which are consistent with the assumptions of FLImP localization fit. This initially partly manual and now fully automated process has been in use and carefully refined by the same team over more than a decade and has always focused on identifying the data and applying analyses which follow the same set of assumptions and has continued to reproduce the same results for the same conditions, apart from improving resolution[1,2,38–41]. The parameters of the final automated approach described here were all fixed a-priori to achieve and refine in our judgement the same process as previously manually performed. This was done before acquisition of any of the data presented in this paper, with the parameters then remaining fixed for all data in the paper, and reproduced results consistent with our previously published work.

1. **Feature detection and tracking** –The previously published single molecule feature detection and tracking software[42] was used to detect and link features through time to produce intensity-position single molecule traces.
2. **Drift determination –** Drift correction was performed using a cross-validation based approach as described in to identify the fiducial bead tracks and use them to determine the drift while rejecting outliers.
3. **Track selection** - Each remaining FLImP video from each dataset was then subject to an automated track selection process to select those tracks and the temporal regions within them which satisfy the assumptions of the photobleaching FLImP model and to which the FLImP model localization fit can be performed.
4. **FLImP localization fit -** The photobleaching fluorophores model was then fitted to the video ROI and temporal regions identified in the track selection process to determine the fluorophore locations for each selected track. Bootstrap resampling was used to obtain robust uncertainties in the locations. An empirical posterior distribution for separation between the fluorophores was calculated for each selected track from the resulting parameters and bootstrap fits.
5. **Measurement quality filtering** - After FLImP localization fitting to each selected track we can applied a set of filters to remove low-resolution, poor-quality results. This process serves to both accelerate and increase the resolution for the decomposition process without compromising it.
6. **1D decomposition -** The population of bootstrap separation measurements for each condition, in the form of a set of empirical posteriors for separation measure $r_{\Delta x}$ (see Interlude: **Separation measure**

below), was then separately decomposed into a model of discrete underlying separation components (see 6. 1D **decomposition** section below)

7. **Posterior comparisons and bootstrap resampling -** To take some account of the finite number of FLImP measurements in each dataset after the measurement quality filter has been applied (typically 100 measurements), we used bootstrap sampling to enable comparison between conditions to consider the significance of the difference between abundance-weighted posteriors between samples.
8. **Multidimensional scaling analysis (MSD)** - To provide an interpretable graphical representation of the difference between bootstrapped 1D-MCMC FLImP posteriors, a dimensionality reduction technique was used produce a 2D representation of the datasets whereby mutations that generated similar 1D-MCMC FLImP posteriors would be in close proximity in the dimensionally reduced space.
9. **2D FLImP triangle pooling** - Extension of the FLImP technique to 2D permits the measurement of trianglular localization sets instead of single separations. Using Wasserstein metric to measure distances between triangles, an agglomerative clustering technique is employed to estimate the number of unique triangles within each 2D FLImP dataset and their associated uncertainties.

Section II. DETAILED FLImP METHODS:

1. Feature detection and tracking -

All single-molecule time series data (for FLImP and single molecule tracking) were initially analyzed using the multidimensional analysis software described previously[42]. Briefly, this software performs frame-by-frame Bayesian segmentation to detect features, then performs a least-squares Gaussian profile fit to locate detected features to sub-pixel precision, then links these features through time to create tracks using a simple proximity-based algorithm. For multichannel data (for colocalization analysis) the software determines cubic polynomial registration transformations from images of fluorescent beads, performs feature detection and tracking independently in each channel before applying the transformations to transform all tracked positions to a common channel.

2. Drift determination

Method to determine drift from a given set of tracks:

- Split tracks at points where their movement between subsequent frames is in the top 5% quantile for that frame. This will reduce the contribution from any mis-tracking, for example a track jumping between close together/overlapping spots.
- Throw out any resulting tracks which last fewer than 20 frames.
- Determine average motion of this resulting set of tracks. This is done by iteratively populating bins of aligned track positions, $\underline{\mathbf{x}}(t) = [x_1(t), x_2(t)\ldots x_{N_t}(t)]$, $\underline{\mathbf{y}}(t) = [y_1(t), y_2(t)\ldots y_{N_t}(t)]$, as a function of time index, t. $\underline{\mathbf{x}}(t)$ and $\underline{\mathbf{y}}(t)$ are initialised to the longest track, and then working through the remaining tracks in order of decreasing length, the next track is shifted in x and y to have the same temporal mean position as $\underline{\mathbf{x}}(t)$ and $\underline{\mathbf{y}}(t)$ at their common time points and then its shifted locations are pooled with those in $\underline{\mathbf{x}}(t)$ and $\underline{\mathbf{y}}(t)$. The final $\underline{\mathbf{x}}(t)$ and $\underline{\mathbf{y}}(t)$ will have multiple values of x and y at each time point from the individual aligned tracks, which are finally averaged at each time point to give an average motion, or drift, of all tracks.

To further mitigate the effect of misidentified beads, and errors in tracking (for example in crowded fields), we use a cross validation approach to identify outlier bead tracks and discard them from our drift calculation. This is performed as follows:

- Identify beads as the 200 tracks lasting at least 80% of duration whose time-averaged brightness is highest. Work only with these from here.
- Repeat 200 times:

- Randomly reject a proportion f_{rej} (=1/3) of the bead tracks, and randomly split remaining $1 - f_{rej}$ of beads into two equal partitions of tracks, A and B.
- Calculate drift as above for each of these partitions separately.
- Calculate RMS residual between partition drift curves after aligning them to have the same mean location in x and y.

- Choose the partition set which gave the lowest RMS residual and pool A and B into one set of bead tracks. The rejected f_{rej} tracks from this partition are likely to have the poorest/worst outlier tracks determined by the cross validation.

- Calculate the drift again for the pooled bead tracks to determine the final drift curve. This was repeated 100 times resampling with replacement which tracks to include to enable frame-by-frame uncertainties to be estimated.

3. Track selection

Each FLImP series typically returned between 1,000 and 10,000 track objects of which only a small fraction was suitable for FLImP analysis. FLImP suitable tracks were defined by the following criteria:

i. In the absence of active fluorophores, track background (the track ROI intensity in the 20 frames beyond the time at which the final fluorophore in a track goes dark) has uniform, zero intensity
ii. Successive fluorophores have additive and approximately equal intensities
iii. Except between sequential frames where level transitions have occurred, fluorophores are stationary in xy position, and their intensity remains constant over time. During transitions, xy positions shift by <0.5 pixels (~60nm).
iv. Fluorophore intensity levels can be interrupted when fluorophores enter transitory non-radiative decay (so-called dark states) including single frame blinking events.
v. A minimum of 5 measurements is required per level for FLImP-fitting

Previously, identification of FLImP suitable tracks was a laborious process, requiring trained operators to manually trawl track lists from each FLImP series to identify tracks that may be suitable for downstream FLImP fitting processes. Recent attempts to automate similar time series analysis for single molecule imaging have relied on Bayesian grouping of localization.[43] While such techniques are indispensable for assigning probabilistic identities to large groups of localizations, as the present 2D FLImP technique typically involves fewer than 10 fluorophores per track object, this enabled simpler, and substantially faster, sequential filtering approach to be used. Such an approach was chosen over emerging 1D convolutional neural networks (1D-CNNs)[44] owing to the tractability of the decision-making process and the lack of a sufficiently large manually labeled training dataset. That said, the outputs of the following sequential filtering approach have been written with this future development in mind by accelerating the generation of labeled training datasets and will be the subject of future work. The authors anticipate that progressing to a 1D-CNN approach is likely to increase the efficiency of FLImP suitable track identification, but at least in the short term this will come at the price of explainability which may limit the ultimate clinical utility of FLImP derived technologies should this path be taken.

The sequential filtering approach to track selection involves passing a population of tracks through a series of filters to rapidly and automatically identify tracks suitable for further FLImP analysis. As tracks with increasing numbers of fluorophores represent nested sets, track subsets may be suitable for further FLImP analysis, even if the entire track is not. As such, a successful automated FLImP suitable track identification algorithm is required to evaluate track suitability at different granularities.

Filters are organized so that the most computationally resource intensive filters are located towards the end of the pipeline, to minimize resource requirement and accelerate this aspect of the process. As such, filtering of a typical FLImP series containing 5000 track objects is typically completed within as little as 60 seconds

using a typical single CPU core and lends itself to parallelization. Furthermore, the track selection process has been written so that it can be applied to tracks containing up to 9 fluorophores (the anticipated limit for a non-Bayesian approach). A detailed description of each filter is provided in the following sections. Filter parameters were developed heuristically on training datasets independent of those presented in the results section of this manuscript and were then held constant throughout. A list of filters is provided below:

i. Label levels - Tracks were first divided into crude intensity levels using the R implementation of the dynamically programmed Optimal k-means clustering algorithm for one dimensional data[45] and the level with the lowest median intensity assigned background (Level 0) and levels with increasing median intensity labeled in ascending numerical order.
ii. Ensure zero background - Remove tracks where the best-fit spot intensity at final location in the track is not zero for the 20 frames after the final time point as determined using a Two One-Sided Test (TOST).[46] The limits of the test were set using 10% of the 95th percentile of track intensity and (α=0.05).
iii. Remove short levels - Next, levels were removed from tracks that contained fewer than 5 frames.
iv. Ensure adequate level intensity separations - Remove tracks with median level intensity separations that are not 2*(level) fold different from the intensity separation between level 1 and background (threshold +- 0.25). This is because we expect fluorophores to have very similar and additive intensities within tracks (but this can vary between tracks).
v. Short inter-level sub-level removal - Sub-levels, defined as sections of track that occur between level-change events whose median intensity is not an integer multiple of the lowest intensity level are identified and those containing fewer than 5 frames are removed from further analysis. These may correspond to either blink states that extend over multiple frames or level transitions that are spread over multiple frames.
vi. Intra-level sub-level removal - Intra-level sub-levels denote the existence of intensity or positional transitions that can be observed within a single level. These were isolated using R change point implementation of the PELT (Pruned Exact Linear time) algorithm to independently detect potential changes of state within each fluorophore level.[47]
vii. Intra-level sub-level positional filter - Intra-level sub-levels are further filtered using Median absolute deviation (MAD)[48] in intensity and x,y positions to remove sub-tracks with MAD >10% of median level intensity and 0.125 pixels respectively as measurements such as these were deemed likely to be too imprecise for FLImP fitting. Background levels were excluded from this filter.
viii. Remove sub-levels with significant intensity gradients and low occupancy - Intra-level and sub-levels containing fewer than 5 frames or with an occupancy (proportion of frames where a spot was detected within the level) <80% were then removed, before all intra-level sub-levels with a significantly non-zero gradient (TOST >5% gradient where α=0.05). Background level was excluded from this filter.
ix. Intra-level sub-level median intensity equivalence - Ensure all intra-level sub-levels have the same median intensity +/- 12.5% using a TOST. This is to ensure levels interrupted by brief transitions between states (i.e. blink states) return to near identical starting conditions so can be classed as the same object. Such level merging increases the efficiency of the track selection process.
x. Inter-level positional shift filter - Remove all sub-tracks that show excessive *x* or *y* positional shifts (median positional shift >0.125 pixels for any sub-track within a level). Excluding background levels.
xi. Minimum frames and levels filter - As quite a few filters have been applied to sub-tracks within the data, ensure that all remaining tracks still contain sufficient frames to be useful for bootstrapping and that all desired sequential levels are still sufficiently represented, depending on the maximum number of levels currently under evaluation. For example, level Background + 1 + 2 for two spot tracks or level Background + 1 + 2 + 3 for three spot tracks).
xii. Spatial neighborhood filter - Remove frames from tracks that are within ~2 PSF (approximately 5 pixels) of another detected fluorophore at any time point. This is because these neighboring objects are likely to contribute to inhomogeneity in the background of the spot of interest (which is currently assumed to be constant that can vary between frames). This is achieved by calculating the cross-nearest neighbor distance for each frame in the selected track for every detected object in the frame.

xiii. Inter-level minimum frames filter - Remove sequential sub tracks that contribute fewer than 5 frames to the track dataset as these were found to be of low quality (i.e., temporally sparse, from small levels etc).

xiv. Identify and remove blinking events - Blinking events, typically single or double frames events that exhibit intensities that are substantially lower than the ordinary intensity distribution of a level. Assuming fluorophore intensity is drawn from a sum of normal distributions for each fluorophore present (n-spots * N(μ,σ)), blinking events can be considered as outliers from this distribution. As such, a modified Z-score,[49] can be used to readily identify and remove outlying intensity, (and localization x and y values) from remaining tracks using the threshold of z<3.

xv. Maximum level filtering and minimum frames per level filtering - Tracks are filtered so that only those containing the desired number of levels are retained. As quite a few filters have been applied to sub-tracks within the data, ensure that all remaining tracks still contain sufficient frames (5 per level) to be useful for bootstrapping and that all desired sequential levels are still sufficiently represented. (level 1 + 2 or level 1 + 2 + 3).

xvi. Split-level matching - To recombine split levels, we need confidence that they are drawn from the same location, here defined as returning a TOST result with limits of +/- 0.1 pixels of x and y where α = 0.05. In rare instances where split levels cannot be merged (i.e., two fluorophores simultaneously switch on and off at the same point), the fluorophore containing the most frames is used.

xvii. Level ordering filter - Split levels of the type lvl1→lvl2→lvl1 within a track can be indicative of two fluorophores (A+B) behaving in one of two ways. A→AB→A or A→AB→B. In the second case, it would be undesirable to merge the two lvl1 subsets. As such, tracks are filtered such that only those with sequentially decreasing levels are selected.

xviii. MAD population intensity filter - A limitation of estimating the intensity of a single fluorophore within each track independently is that this method will not distinguish between situations where multiple fluorophores go dark at once. For example, the two step tracks; for the two-fluorophore system: AB-B-0 and the four-fluorophore system: ABCD→AB→0 may both exhibit 2N→N→0 intensity steps. While the second case is likely to be rare, this eventuality can only be eliminated by considering the fluorophore intensity at the population scale. Here, the intensity of each fluorophore is pooled and tracks excluded when fluorophores were found to possess MAD values exceeding 2.5 of the fluorophore population.

4. FLImP localization fit

As described in[41], the FLImP fitting process determines the locations of the two molecules in each track and their uncertainties by fitting a model of two overlapping, photobleaching Gaussian fluorophore PSF profiles to the ROI of the spot during the frames identified by the track selection procedure (described above). Briefly, a 7-parameter least square fit is performed, where the parameters are the intensity and x and y location of the two fluorophores and their common PSF size. Which fluorophores to include in the model at each time point is identified as part of the track selection procedure. The drift of the sample is accounted for in the fitting process, by assuming the fluorophores move in time following the drift while remaining fixed with respect to one another. The fitted profiles of other nearby features in the ROI detected by the feature detection are removed from the ROI images before this FLImP fit is performed to remove their contribution. By repeating the fit 2400 times, each time resampling with replacement from the $n_x \times n_y \times n_t$ intensity values (the unravelled ROI-time image volume), we obtain an empirical estimate of the uncertainties in the parameters. From this, we can obtain an empirical probability distribution, or posterior given the data, for the fitted parameters and anything which can be calculated from them, such as the fluorophore separation. We showed in[41] how this approach provides robust estimates, including that of the uncertainties, without the need for an explicit model for image noise.

5. Measurement quality filtering

Datasets of FLImP measurements for a particular condition typically contain many hundreds or even thousands of individual single molecule track measurements with a range of localisation error and other qualities. Beyond the filtering for suitable tracks for FLImP analysis described in 'Track selection' (see above)

after the FLImP fit to each track selection has been performed we can further narrow down the dataset to good quality results by applying a set of filters based on properties of the fitted measurements. By removing low resolution, poor quality results we speed up decomposition and other analysis of the FLImP measurements to increase the resolution for the decomposition without compromising it. We filter them as follows:

i. Only keep measurements whose separation between determined fluorophore locations is $r < 70$ nm. We are not interested in longer separations.

ii. Only keep measurements for which the 69% confidence interval (CI) in $r_{\Delta x} < 8$nm. This means only high localization precision measurements are used. Note this filter uses the $r_{\Delta x}$ CI which is not correlated with separation, rather than the $|r_{\Delta x}|$ separation which is. This ensures this filter does not bias the resulting separation distribution.

iii. Measurements where the localization posterior of either fluorophore has too high an asymmetry are rejected as this may be a sign of a poor measurement. The ratio of the minor axis to the major axis of the (x, y) location posterior covariance ellipse must be > 0.5 for both spots in the measurement.

iv. Only include selected track frame ranges which follow the sequence 2 fluorophores → 1 fluorophore → no fluorophores, not any which increase to 2 or more fluorophores before dropping again. This reduces the chance of misinterpreting a switch from for example fluorophore A → A+B → B as A → A+B → A and thus incorrectly measuring the separation.

v. Finally, of the measurements that pass the above filters we keep the 100 highest resolution measurement, with resolution defined as the 69% $r_{\Delta x}$ CI.

Interlude: Separation measure

The difference in position, or separation, between locations is normally represented by the L2 norm between the locations. When those locations are uncertain, the probability distribution for their separation also has uncertainty and can take forms which are problematic for measuring short separations in particular. Measuring separations between uncertain locations is a common task in single molecule imaging. In the simplest case, where the uncertainty in each location can be represented by the same axisymmetric 2D normal distribution, the probability distribution for the L2 separation will be a Rician distribution. In the extreme case that the locations are coincident, i.e., the true separation is zero, the Rician distribution will have zero probability at separation zero. In general, using the L2 norm means low sensitivity and possible biases in measurement of separations of order the location uncertainty or less. In traditional 1D-FLImP (which used Ricean modelling of the L2 norms) this meant separations <8nm could not be accurately quantified.

We have used an alternative measure in some areas of our new FLImP analysis which we call $r_{\Delta x}$. $r_{\Delta x}$ is calculated as follows:

i. A pair of emitters, A and B, have locations in space $\underline{\mathbf{R}_A}$ and $\underline{\mathbf{R}_B}$ described by 2D probability distributions $\Pr(\underline{\mathbf{R}_A})$ and $\Pr(\underline{\mathbf{R}_B})$ respectively (e.g., from the 2400 FLImP bootstrap location samples for each emitter in the track object.
ii. The $\underline{\mathbf{L}}$ direction is defined as the direction from the estimated location of A, $\langle\underline{\mathbf{R}_A}\rangle$ to the estimated location of B, $\langle\underline{\mathbf{R}_B}\rangle$, i.e. $L = (\langle\underline{\mathbf{R}_B}\rangle - \langle\underline{\mathbf{R}_A}\rangle)/|\langle\underline{\mathbf{R}_B}\rangle - \langle\underline{\mathbf{R}_A}\rangle|$.
iii. The separation measure used, $r_{\Delta x}$, between two points $\underline{\mathbf{r}_A}$ and $\underline{\mathbf{r}_B}$ chosen from emitter posteriors $\Pr(\underline{\mathbf{R}_A})$ and $\Pr(\underline{\mathbf{R}_B})$ respectively is the vector between those two points resolved parallel to $\underline{\mathbf{L}}$, i.e. $r_{\Delta x} = (\underline{\mathbf{r}_B} - \underline{\mathbf{r}_A}).\underline{\mathbf{L}}$. $r_{\Delta x}$ can be positive or negative, and has the desirable property that in the event that $\Pr(\underline{\mathbf{R}_A})$ and $\Pr(\underline{\mathbf{R}_B})$ are axisymmetric Gaussians the probability distribution of $r_{\Delta x}$ between A and B, $\Pr(r_{\Delta x})$, will be a Gaussian centred on $|\,\underline{\mathbf{R}_B} - \underline{\mathbf{R}_A}\,|$. It does not suffer from the insensitivity or bias at short separations suffered by the L2 norm and can in principle even measure zero separations.
iv. Normal distribution-based approximations to $\Pr(r_{\Delta x})$ are used in our 1D-FLImP decomposition which allows fast evaluation.

6. 1D decomposition

FLImP measurements as posteriors

We have n spots (tracks) (labeled $i = 1..n$) for each of which we have measured a single separation from the FLImP localization fit. We interpret the bootstrap distribution for separation x from spot i given its data D_i as a posterior for the separation, $\Pr(x|D_i)$. From Bayes' Theorem,

$$\Pr(x|D_i) = \frac{\Pr(D_i|x)\Pr(x)}{\Pr(D_i)}$$

If we limit x to the range 0 to x_{max} with a uniform prior, then in this range we have

$$\Pr(x) = \frac{1}{x_{max}}$$

We can then write the likelihood for D_i given x,

$$\Pr(D_i|x) = \Pr(x|D_i)\Pr(D_i)(x_{max}) = a_i \Pr(x|D_i)$$

Equation 1

where $a_i = x_{max}\Pr(D_i)$.

Decomposition model

For our model we assume the structure being measured has N distinct components, $k = 1..N$, each component having a different separation, $\underline{\mathbf{x}} = \{x_1,..,x_N\}$. Each FLImP separation corresponds to one of these components or is a spurious "clutter" measurement. This assignment for each measurement is denoted by $\underline{\mathbf{K}} = \{k_1,..k_n\}$, $k_i = 1..N$, where measurement i corresponds to component k_i with separation x_{k_i}, and $k_i = 0$ means measurement i is clutter.

Assuming the model components are precise, and the separation of clutter components is uniformly distributed in the x domain, then the probability distribution of separation for component k is

$$\Pr(x|k) = \begin{cases} \delta(x - x_k) & \text{if } k > 0 \\ 1/\Delta x & \text{if } k = 0 \text{ and } x \text{ is within the domain} \\ 0 & \text{if } k = 0 \text{ and } x \text{ is not within the domain} \end{cases}$$

Equation 2

where δ is the Dirac delta function and Δx is the size of the domain of x.

We assume that the probability of the measurement process yielding a clutter measurement is P_c, so that typically this should be the proportion of measurements which is clutter. We assume that the non-clutter components are all equally probable.

We therefore have model parameters $\{\underline{\mathbf{K}}, \underline{\mathbf{x}}, P_c\}$.

Model likelihood and posterior

Each spot measurement is completely independent, so that the likelihood for the entire set of measurements $\underline{\mathbf{D}} = \{D_1,..,D_n\}$ given $\underline{\mathbf{x}}$ and $\underline{\mathbf{K}}$ is

$$\Pr(\underline{\mathbf{D}}|\underline{\mathbf{K}}, \underline{\mathbf{x}}) = \prod_{i=1}^{n} \Pr(D_i|k_i, \underline{\mathbf{x}})$$

We have $\Pr(D_i|x)$ from Equation 1 and $\Pr(x|k)$ from Equation 2, which we use to calculate $\Pr(D_i|k_i, \underline{\mathbf{x}})$. P_c does not figure here as once $\underline{\mathbf{x}}$ and each k_i are given the likelihood of the data is known.

$$\Pr\left(D_i|k_i,\underline{\mathbf{x}}\right) = \int_{-\infty}^{\infty}\Pr(D_i|x)\Pr(x|k_i)\,dx$$

$$= \begin{cases} \int_{-\infty}^{\infty}\Pr(D_i|x)\delta\left(x-x_{k_i}\right)dx = \Pr\left(D_i|x=x_{k_i}\right) \\ \qquad = a_i\Pr\left(x_{k_i}|D_i\right) \quad \text{if } k_i>0 \\ \int_{-\infty}^{\infty}\Pr(D_i|x)/\Delta x dx = \int_{-\infty}^{\infty} a_i\Pr(x|D_i)/\Delta x dx \\ \qquad = a_i/\Delta x \quad \text{if } k_i=0 \end{cases}$$

We abbreviate $L_{k_i} = \Pr\left(D_i|k_i,\underline{\mathbf{x}}\right)/a_i$ so that

$$L_{k_i} = \begin{cases}\Pr\left(x_{k_i}|D_i\right) & \text{if } k_i>0 \\ 1/\Delta x & \text{if } k_i=0\end{cases}$$

Equation 3

so that

$$\Pr\left(\underline{\mathbf{D}}|\underline{\mathbf{K}},\underline{\mathbf{x}}\right) = \prod_{i=1}^{n}\Pr\left(D_i|k_i,\underline{\mathbf{x}},P_c\right) = \prod_{i=1}^{n} a_iL_{k_i} = A\prod_{i=1}^{n}L_{k_i}$$

where $A=\prod_{i=1}^{n}a_i$.

From Bayes theorem we have:

$$\Pr\left(\underline{\mathbf{K}},\underline{\mathbf{x}}|\underline{\mathbf{D}}\right) = \frac{\Pr\left(\underline{\mathbf{D}}|\underline{\mathbf{K}},\underline{\mathbf{x}}\right)\Pr\left(\underline{\mathbf{K}},\underline{\mathbf{x}}\right)}{\Pr\left(\underline{\mathbf{D}}\right)} = \frac{\Pr\left(\underline{\mathbf{D}}|\underline{\mathbf{K}},\underline{\mathbf{x}}\right)\Pr\left(\underline{\mathbf{x}}\right)}{\Pr\left(\underline{\mathbf{D}}\right)} = \frac{\Pr\left(\underline{\mathbf{x}}\right)}{\Pr\left(\underline{\mathbf{D}}\right)}A\prod_{i=1}^{n}L_{k_i}$$

as the prior $\Pr\left(\underline{\mathbf{K}},\underline{\mathbf{x}}\right) = \Pr\left(\underline{\mathbf{K}}\right)\Pr\left(\underline{\mathbf{x}}\right) = \Pr\left(\underline{\mathbf{x}}\right) = \prod_{k=1}^{N}\Pr(x_k) = \prod_{k=1}^{N}\pi_{x_k}$, $\underline{\mathbf{K}}$ is only constrained through $\Pr\left(\underline{\mathbf{K}}|P_c,\underline{\mathbf{D}}\right)$ (see below) so $\Pr\left(\underline{\mathbf{K}}\right)=1$ and we assume a uniform prior on x within the domain

$$\Pr(x_k) = \pi_{x_k} = \begin{cases}1/\Delta x & \text{if } x \text{ in domain} \\ 0 & \text{if } x \text{ outside domain}\end{cases}$$

Our model for clutter gives a probability for the assignments, $\Pr\left(\underline{\mathbf{K}}|P_c,\underline{\mathbf{D}}\right)$. This is composed of two components as follows.

First, the number of measurements assigned to clutter, $n_c=\sum_{i=1}^{n}\delta_{k_i,0}$ where δ is the Kronecker delta, will be binomially distributed with probability P_c, i.e.

$$\Pr(n_c) = \frac{n_c!}{n!\,(n-n_c)!}P_c^{n_c}(1-P_c)^{n-n_c}$$

Secondly, the assumption that the clutter is uniformly distributed in the x domain can be expressed as a multinomial prior on the number of measurements assigned to clutter in a set of B equal-sized bins $b=1..B$ in the x domain, $\underline{\mathbf{n}_{c_b}}=\{n_{c_1}..n_{c_B}\}$. We have $n_{c_b}=\sum_{i=1}^{n}\delta_{k_i,0}\,F(b,\widehat{x_i})$ where

$$F(b,x) = \begin{cases}1 & \text{if } (b-1)\Delta x/B \le x < b\Delta x/B \\ 0 & \text{otherwise}\end{cases}$$

and $\widehat{x_i}$ is $\langle\Pr(r_{\Delta x}|D_i)\rangle$, the mean $r_{\Delta x}$ for measurement i. We use $B=20$ bins. So

$$\Pr\left(\underline{\mathbf{n}_{c_b}}\right) = \frac{n_c!}{\prod_{b=1}^{B}n_{c_b}!}\prod_{b=1}^{B}(1/B)^{n_{c_b}}$$

This gives:

$$\Pr(\underline{\mathbf{K}}|P_c, \underline{\mathbf{D}}) = \begin{cases} \Pr(n_c) & \text{if } n_c = 0 \\ \Pr(n_c)\Pr\left(\underline{\mathbf{n}_{c_b}}\right) & \text{if } n_c > 0 \end{cases}$$

The posterior probability can be separated into two components,

$$\Pr(\underline{\mathbf{K}}, \underline{\mathbf{x}}, P_c|\underline{\mathbf{D}}) = \Pr(\underline{\mathbf{K}}, \underline{\mathbf{x}}|\underline{\mathbf{D}})\Pr(\underline{\mathbf{K}}, P_c|\underline{\mathbf{D}})$$

We can write

$$\Pr(\underline{\mathbf{K}}, P_c|\underline{\mathbf{D}}) = \Pr(\underline{\mathbf{K}}|P_c, \underline{\mathbf{D}})\Pr(P_c|\underline{\mathbf{D}}) = \Pr(\underline{\mathbf{K}}|P_c, \underline{\mathbf{D}})\Pr(P_c) = \Pr(\underline{\mathbf{K}}|P_c, \underline{\mathbf{D}})\pi_{P_c}$$

since the clutter probability does not depend on the data. For the clutter fraction prior we assume a beta distribution $\Pr(P_c) = \pi_{P_c} = \text{Beta}(P_c, \alpha, \beta)$ with shape parameters $\alpha = 1, \beta = 9$ which peaks at $P_c = 0.0$ and has $\langle \pi_{P_c} \rangle = 0.1$.

We therefore have the full posterior

$$\Pr(\underline{\mathbf{K}}, \underline{\mathbf{x}}, P_c|\underline{\mathbf{D}}) = \frac{\Pr(\underline{\mathbf{K}}|P_c, \underline{\mathbf{D}})\pi_{P_c}\Pr(\underline{\mathbf{D}}|\underline{\mathbf{K}}, \underline{\mathbf{x}})\Pr(\underline{\mathbf{x}})}{\Pr(\underline{\mathbf{D}})} = \frac{\Pr(\underline{\mathbf{K}}|P_c, \underline{\mathbf{D}})\pi_{P_c}}{\Pr(\underline{\mathbf{D}})} A \prod_{k=1}^{N} \pi_{x_k} \prod_{i=1}^{n} L_{k_i}$$

Equation 4

Sampling from the posterior

Our model parameters are separations $\underline{\mathbf{x}}$, clutter probability P_c and assignments $\underline{\mathbf{K}}$. We wish to draw a representative set of samples of these from the posterior $\Pr(\underline{\mathbf{K}}, \underline{\mathbf{x}}, P_c|\underline{\mathbf{D}})$. We do this using the Metropolis-Hastings Markov Chain Monte Carlo (MCMC) sampling approach, sampling parameters in turn as follows:

1. Initialise parameters at iteration $t = 0$. Each component of $\underline{\mathbf{x}}(t = 0)$ is sampled independently from π_{x_k}, $P_c(t = 0)$ is sampled from the clutter prior π_{P_c} and each component k_i of assignment $\underline{\mathbf{K}}(t = 0)$ is sampled independently from $0..N$ with a uniform distribution.

2. For each iteration $t \rightarrow t + 1$:

   a. Metropolis-Hastings sample $P_c(t + 1)$ given $\underline{\mathbf{x}}(t)$ and $\underline{\mathbf{K}}(t)$. The proposal distribution for $P_c(t + 1)$ is $\mathcal{N}(P_c(t), \sigma_{P_c}^2)$ limited to the range $0..1$ with $\sigma_{P_c} = 0.1$.

   b. Jointly Metropolis-Hastings sample $\underline{\mathbf{x}}(t + 1)$ and $\underline{\mathbf{K}}(t + 1)$ given $P_c(t + 1)$ by Metropolis-Hastings sampling each component x_k of $\underline{\mathbf{x}}$ $(k = 1..N)$ in turn, jointly with $\underline{\mathbf{K}}$. Perform $j = 1..N$ sub-iterations yielding in turn updated parameters $\underline{\mathbf{x}}'(j)$ and $\underline{\mathbf{K}}'(j)$, starting with $\underline{\mathbf{x}}'(0) = \underline{\mathbf{x}}(t)$ and $\underline{\mathbf{K}}'(0) = \underline{\mathbf{K}}(t)$, with each iteration as follows:

      i. Proposal for $\underline{\mathbf{x}}'(j)$ is $\underline{\mathbf{x}}^*$, with component $x_{k=j}^*$ from proposal distribution $Q\left(x_{k=j}^*|x_{k=j}(t)\right)$ and components $x_{k\neq j}^* = x'_k(j-1)$. $Q(x'|x) = 0.5 * \mathrm{P_{DATA}}(x') + 0.5 * \left(w_1\mathcal{N}(x' - x, \sigma_1^2) + w_2\mathcal{N}(x' - x, \sigma_2^2)\right)$ with $(w_1, \sigma_1, w_2, \sigma_2) = (0.7,2\text{nm}, 0.3,5\text{nm})$. $\mathrm{P_{DATA}}(x) = \frac{1}{n}\sum_{i=1}^{n} \Pr(x|D_i)$ is the normalised sum of sample data posteriors (see FLImP measurements as posteriors).

      ii. Proposal $\underline{\mathbf{K}}^*$ for $\underline{\mathbf{K}}'(j)$ has each component directly sampled from Equation 6 given the proposed $\underline{\mathbf{x}}^*$.

iii. Metropolis-Hastings sample to accept/reject a move from $\underline{\mathbf{x}}'(j-1)$, $\underline{\mathbf{K}}'(j-1)$, $P_c(t+1)$ to $\underline{\mathbf{x}}'(j)$, $\underline{\mathbf{K}}'(j)$, $P_c(t+1)$. Set $\underline{\mathbf{x}}'(j)$, $\underline{\mathbf{K}}'(j)$ to $\underline{\mathbf{x}}'(j-1)$, $\underline{\mathbf{K}}'(j-1)$ if the move is rejected, and to $\underline{\mathbf{x}}^*$, $\underline{\mathbf{K}}^*$ if it is accepted.

c. After the sub-iterations we keep $\underline{\mathbf{x}}(t+1) = \underline{\mathbf{x}}'(N)$ and $\underline{\mathbf{K}}(t+1) = \underline{\mathbf{K}}'(N)$.

Jointly sampling $\underline{\mathbf{x}}$ components and $\underline{\mathbf{K}}$ this way ensures a sufficient acceptance rate by proposing changes in assignment commensurate with changes in component locations.

Each new $\underline{\mathbf{x}}$ sample is sorted in ascending order.

Sampling is performed until convergence, and samples are thinned to keep every 20th sample (autocorrelation tests on sample chains reveal this thinning to be sufficient). The first 50% of samples are discarded to exclude the burn-in period. Two independent chains of samples are run, each initialized separately, and convergence is checked periodically after each chain has drawn the same number of samples comparing the second 50% of thinned samples in each chain using the Gelman-Rubin test. The test is initially performed after 2000 iterations (giving 100 post-burnin thinned samples). If the test fails sampling resumes to increase the iterations by 50% (i.e., to 3000, 4500... iterations). The sampling fails if convergence isn't reached after 40000 iterations (10000 post-burnin thinned samples). The chains are considered to have converged if $|1-\hat{R}| < 0.001$ for each of x_k ($k = 1..N$) and P_c compared separately between the two chains (A and B), where $\hat{R} = \sqrt{\frac{\hat{\sigma}^2}{W}}$ is the univariate Gelman-Rubin statistic[50] for each parameter, and

$$W = \frac{\sigma_A^2 + \sigma_B^2}{2}$$

$$B = n_{iter}((\mu_A - \mu)^2 + (\mu_B - \mu)^2)$$

$$\hat{\sigma}^2 = \frac{n_{iter} - 1}{n_{iter}} W + \frac{1}{n_{iter}} B$$

with n_{iter} samples used from each chain, the variance of the samples from each chain is $\sigma_{A/B}^2$ respectively, the mean of the samples in each chain is $\mu_{A/B}$ and $\mu = (\mu_A + \mu_B)/2$. Of the two chains for each N we adopt the one with the lowest $AVBIC$ (Average Bayesian Information Criterion, see 'Model selection' for further analysis (they are almost identical in all cases given the stringent convergence criterion).

We use $0..\Delta x$ as the domain of our separation measurement, with Δx set so that all of the probability in $\mathrm{P_{DATA}}(x)$ is in the domain.

We use $x = |r_{\Delta x}|$ (see 'Interlude: **Separation measure**') for our separation measure in the decomposition. For computational efficiency we replace each measurement posterior $\Pr(r_{\Delta x}|D_i)$ with a Gaussian approximation (from its mean and variance).

<u>*Assignment proposal distribution*</u>

While the assignments do have a joint posterior due to the inclusion of clutter probability in the model, we ignore this and treat each measurement separately when calculating the proposal distribution for $\underline{\mathbf{K}}$. This should be a good approximation for the target distribution for typical P_c. We calculate the probability $\Pr(k_i|D_i, \underline{\mathbf{x}}, P_c)$ of a particular assignment k_i for measurement i. From Bayes theorem:

$$\Pr(k_i|D_i, \underline{\mathbf{x}}, P_c) = \frac{\Pr(D_i|k_i, \underline{\mathbf{x}}, P_c)\Pr(k_i|\underline{\mathbf{x}}, P_c)}{\Pr(D_i|\underline{\mathbf{x}}, P_c)} = \frac{\Pr(D_i|k_i, \underline{\mathbf{x}}, P_c)\Pr(k_i|\underline{\mathbf{x}}, P_c)}{\sum_{k_i=0}^{N} \Pr(D_i|k_i, \underline{\mathbf{x}}, P_c)\Pr(k_i|\underline{\mathbf{x}}, P_c)}$$

Equation 5

We assume that all non-clutter assignments have equal prior probability giving priors for k

$$\Pr(k|P_c, N) = \pi_k = \begin{cases} P_c & \text{if } k = 0 \\ (1-P_c)/N & \text{if } k = 1..N \end{cases}$$

We therefore have from Equation 5 and Equation 3

$$\Pr\left(k_i|D_i, \underline{\mathbf{x}}, P_c\right) = \frac{a_i L_{k_i}\pi_{k_i}}{\sum_{k_i=0}^{N} a_i L_{k_i}\pi_{k_i}} = \frac{L_{k_i}\pi_{k_i}}{\sum_{k_i=0}^{N} L_{k_i}\pi_{k_i}}$$

Equation 6

Model selection

To choose an appropriate number of components to fit to a particular dataset we repeat the Metropolis-Hastings parameter estimation for N from 1 to 9 and then score each fit with the $AVBIC$, over the parameter samples[51]

$$AVBIC = n_{par}\ln n - \frac{1}{n_{samples}}\sum_{i_{sample}=1}^{n_{samples}} 2\ln\Pr\left(\underline{\mathbf{K}}|P_c, \underline{\mathbf{D}}\right)\pi_{P_c}\Pr\left(\underline{\mathbf{D}}|\underline{\mathbf{K}}, \underline{\mathbf{x}}\right)\Pr\left(\underline{\mathbf{x}}\right)$$

where $n_{par} = 1 + N + n$ is the total number of parameters being optimised for P_c, $\underline{\mathbf{x}}$ and $\underline{\mathbf{K}}$ respectively and $\Pr\left(\underline{\mathbf{K}}|P_c, \underline{\mathbf{D}}\right)\pi_{P_c}\Pr\left(\underline{\mathbf{D}}|\underline{\mathbf{K}}, \underline{\mathbf{x}}\right)\Pr\left(\underline{\mathbf{x}}\right)$ is from Equation 4. The Bayesian Information Criterion is an approximation to the hard-to-calculate Bayesian evidence for comparing different model fits while avoiding overfitting, and the average BIC modifies this to apply to MCMC results. We choose the smallest N for which $AVBIC$ is at least 10 lower than that for all smaller N, i.e. the simplest decomposition (fewest components) which is demanded by the data according to $AVBIC$.

Using the Metropolis-Hastings posterior samples

The Metropolis-Hastings process produces a representative set of samples from the full posterior distribution, which will explore uncertainties in all parameters, including in the locations of the separation components and correspondence between measurements and individual components. Before sorting, the components in $\underline{\mathbf{x}}$ from one sample to the next need not correspond to one another (it is a Markov chain of samples). Even after sorting in separation they may not correspond to one another from one sample to the next. When visualising and taking estimates and confidence intervals we wish to reorder these components between samples so that they correctly correspond as well as possible. This is done by reordering the components in $\underline{\mathbf{x}}$ independently in each Metropolis-Hastings sample to best match the order in the best (most probable) sample, $\underline{\mathbf{K}}_{best\ sample}$, as follows.

1. Use the measurement assignments $\underline{\mathbf{K}}_{best\ sample}$, determine the normalised sum of sample data posteriors (see 'FLImP measurements as posteriors') separately for each component, i.e. $\mathrm{P}_{\mathrm{DATA},k}(x) = \frac{\sum_{i=1}^{n}\delta_{k_i,k}\Pr(x|D_i)}{\sum_{i=1}^{n}\delta_{k_i,k}}$ where δ is the Kronecker delta.

2. For each sample, consider all possible permutations of $\underline{\mathbf{x}}_{sample}$, scoring each permutation $\underline{\mathbf{x}}_{sample}{}'$ with a probability $P' = \prod_{k=1}^{n}\mathrm{P}_{\mathrm{DATA},k}\,(x_k{}')$. The best permutation for this sample will be that which maximises P'.

3. To allow that some peaks are less clear than others, this process was repeated, but finding for each sample the reordered subset of $N - N_{ignore}$ components of $\underline{\mathbf{x}}_{sample}$ which best matches the narrowest $N - N_{ignore}$ components of $\mathrm{P}_{\mathrm{DATA},k}(x)$, and sorting the ignored components by x. This is performed for $N_{ignore} = 1,2,..N-2$.

4. Steps 2 and 3 yield several proposed relabelings of components between samples (for $N_{ignore} = 0,1,2,..N-2$). Whichever set yields components whose posterior sample distributions in x, $\Pr(x_k)$

least overlap with one another is chosen as the best relabeling and adopted for interpretation of results. The overlap between components j and l is defined as

$$Overlap_{j,l} = \int_0^{\Delta x} n_j \,\mathrm{Pr}(x_j|D) n_l \mathrm{Pr}(x_l|D) dx$$

where n_j and n_l are the number of measurements assigned to these components in the most probable sample ($n_{best,k} = \sum_{i=1}^{n} \delta_{k_{best\,sample,i}, k}$, so weighting overlap by abundance of contributing components). The overlap to be minimised is the summed overlap of all pairs of components, $Overlap = \sum_{j=1}^{N} \sum_{l=j+1}^{N} Overlap_{j,l}$.

After this process we have posterior samples from which clear estimates for separate components can be obtained when the data supports this, and where some components are not clearly resolved this will be clear with large ranges of x for some components.

We are most interested in the distribution of separations revealed by these decompositions, and the proportion of measurements attributed to each, and to extract various estimates from the posterior distribution samples. Our plots, such as Fig. 3b, main text, show the posterior distributions of x weighted by $f_{median,k}$, the median proportion of measurements assigned to each component, $f_{median,k}\mathrm{Pr}(x_k)$, coloured separately for each component. The grey background in each plot is $\mathrm{P_{DATA}}(x)$. In the legend for each plot is the median proportion of measurements assigned to clutter, the median $\mathrm{Pr}(x_k|D)$ and limits of its most compact 68% confidence interval. Underneath each plot is a point showing the individual FLImP measurement locations ($|\mathrm{Pr}(r_{\Delta x}|D_i)|$, coloured according to their model assignment, with clutter assignments in black.

We also define the sum of abundance-weighted posteriors of each component, $\mathrm{P_w}(x|D) = \sum_{k=1}^{N} f_{median,k} \,\mathrm{Pr}(x_k|D)$. This ignores the assignment of each component and just asks how much evidence the decomposition gives for the presence of each separation in the sample.

7. Posterior comparisons and bootstrap resampling

To compare and contrast the decomposition of different FLImP datasets, we compare the abundance-weighted posteriors, $\mathrm{P_w}(|r_{\Delta x}|\,|D)$, between samples. To take some account of the finite number of FLImP measurements in each dataset, which is typically $n = 100$ measurements after the measurement quality filter has been applied, we use bootstrap sampling. For each dataset we created 20 datasets all of size n by bootstrap resampling from the n measurements in the true dataset. We repeat the Metropolis-Hastings decomposition process separately on each of these, for the number of components, N determined from the $AVBIC$ test on the true (unresampled dataset). For each of these we also calculate $\mathrm{P_w}(|r_{\Delta x}|\,|D)$. The variation between between these curves at each x gives a measure of the uncertainty due to the finite number of measurements in the distribution. In plots such as Fig. 3e (main text) we show $\mathrm{P_w}(|r_{\Delta x}|\,|D)$ for the true data as a solid line and an error band showing the 20%–80% percentiles of the bootstrap sample distribution of $\mathrm{P_w}(|r_{\Delta x}|\,|D)$ as a function of $|r_{\Delta x}|$. This enables comparison between conditions to consider the significance of difference between distributions.

8. Multidimensional scaling analysis (MDS)

Distances between each pair of bootstrapped 1D-MCMC FLImP posteriors were measured using the Wasserstein metric,[8] whereby identical histograms would have a Wasserstein metric of zero and more different histograms would exhibit larger positive values. As Wasserstein metric satisfies the triangle inequality,[9] these distances were used to construct a distance matrix between all pairs of histograms before the Multidimensional scaling (MDS)[52]. The dimensionality reduction technique was applied to this distance matrix to produce a simplified visualization of the similarity of the conditions investigated. SCREE plot evaluation via heuristic breakpoint detection suggests the majority of the variability in the dataset could be described by two principal components and conditions that produce more similar histograms of separations would appear more closely together in the plot. As Wasserstein metric does not provide information regarding

the direction of the change (whether oligomers are getting larger or smaller between conditions), plots were overlay with a vector denoting the directionality of increasing oligomer size, defined as an increasing fraction of the FLImP histogram that comprised separations greater than 20 nm. An estimate of the uncertainty in the position of each condition within the MDS plot was obtained by including 1D-MCMC FLImP posteriors derived from 20 bootstrap resamplings of the 1D-FLImP datasets for each condition in the calculation of distance matrix and subsequent MDS analysis, fixing the number of components in each case apriori to that obtained when fitting the entire dataset. This analysis permitted the addition of confidence ellipses to the MDS plots that represent the 95% confidence interval for each condition. Here, the highlighted centre of each ellipse is taken as the 1D-MCMC FLImP posteriors derived from fitting the entire (non-bootstrapped) dataset.

9. 2D FLImP Triangle Pooling

2D FLImP is an extension of 1D FLImP whereby FLImP measurements are extended to include three fluorophores per measurement object. As the number of fluorophores present within FLImP tracks represent a nested set, meaning the same track selection criteria that is used for 1D FLImP can be trivially extended to the 2D case containing three or more fluorophore objects. Depending on labelling conditions, 2D FLImP tracks can be rarer than 1D FLImP measurements. 2D FLImP imaging returns a population of triangles, providing information about the separation of a group (three or more) of fluorescently labelled locations in the sample of interest. Our interpretation assumes that multiple FLImP measurements can be treated as a representative sample from all of the possible separation combinations between labelled locations in the experimental sample. Distances between each pair of bootstrapped 2D-MCMC FLImP posteriors were measured using the Wasserstein metric,[8] whereby identical histograms would have a Wasserstein metric of zero and more different histograms would exhibit larger values. As Wasserstein metric satisfies the triangle inequality.[9] Assuming there is a finite, discrete and precise set of separations between labelled locations in our structure(s), we wish to infer from our 2D FLImP measurements the number of these separations, their value and uncertainties and relative position in 2D space. Optimally exploiting the many highly quantitative 2DFLImP measurements and using the revised separation measure, *dx*, described elsewhere, should give more robust, precise, and unbiased structural information down to zero separation. The Distance matrix of triangle-relatedness was used to construct a dendrogram with optimal cuts determined using Bayesian Hierarchical clustering approach as described in,[10] The resulting grouped triangles were optimally aligned before pooling as illustrated in Supplementary Fig. 5d-5j.

Supplementary References

1. Zanetti-Domingues, L. C. *et al.* The architecture of EGFR's basal complexes reveals autoinhibition mechanisms in dimers and oligomers. *Nat. Commun.* **9**, 4325 (2018).
2. Needham, S. R. *et al.* EGFR oligomerization organizes kinase-active dimers into competent signalling platforms. *Nat. Commun.* **7**, 13307 (2016).
3. Macdonald-Obermann, J. L. & Pike, L. J. Allosteric regulation of EGF receptor ligand binding by tyrosine kinase inhibitors. *J. Biol. Chem.* **293**, 13401–13414 (2018).
4. Defize, L. H. *et al.* Signal transduction by epidermal growth factor occurs through the subclass of high affinity receptors. *J. Cell Biol.* **109**, 2495–2507 (1989).
5. Iyer, R. S. *et al.* Drug-resistant EGFR mutations promote lung cancer by stabilizing interfaces in ligand-free kinase active EGFR oligomers. Zenodo https://doi.org/10.5281/zenodo.10567249 (2024).
6. Arkhipov, A. *et al.* Architecture and Membrane Interactions of the EGF Receptor. *Cell* **152**, 557–569 (2013).
7. Bucevičius, J., Lukinavičius, G. & Gerasimaitė, R. The Use of Hoechst Dyes for DNA Staining and Beyond. *Chemosensors* **6**, 18 (2018).
8. Vaserstein, L. N. Markov processes over denumerable products of spaces, describing large systems of automata. *Ross. Akad. Nauk Probl. Peredachi Informatsii* **5**, 64–72 (1969).
9. Clement, P. & Desch, W. An elementary proof of the triangle inequality for the Wasserstein metric. *Proc. Am. Math. Soc.* **136**, 333–339 (2008).
10. Heller, K. A. & Ghahramani, Z. Bayesian hierarchical clustering. in *Proceedings of the 22nd international conference on machine learning* 297–304 (Association for Computing Machinery, New York, NY, USA, 2005). doi:https://doi.org/10.1145/1102351.1102389.
11. Zhang, X., Gureasko, J., Shen, K., Cole, P. A. & Kuriyan, J. An allosteric mechanism for activation of the kinase domain of epidermal growth factor receptor. *Cell* **125**, 1137–49 (2006).

12. Yoshikawa, S. *et al.* Structural basis for the altered drug sensitivities of non-small cell lung cancer-associated mutants of human epidermal growth factor receptor. *Oncogene* **32**, 27–38 (2013).

13. Kovacs, E. *et al.* Analysis of the role of the C-terminal tail in the regulation of the epidermal growth factor receptor. *Mol. Cell. Biol.* **35**, 3083–3102 (2015).

14. Wang, J. *et al.* Quantifying EGFR Alterations in the Lung Cancer Genome with Nanofluidic Digital PCR Arrays. *Clin. Chem.* **56**, 623–632 (2010).

15. Zhang, F. *et al.* Quantification of Epidermal Growth Factor Receptor Expression Level and Binding Kinetics on Cell Surfaces by Surface Plasmon Resonance Imaging. *Anal. Chem.* **87**, 9960–9965 (2015).

16. Casalino, L. *et al.* Beyond Shielding: The Roles of Glycans in the SARS-CoV-2 Spike Protein. *ACS Cent. Sci.* **6**, 1722–1734 (2020).

17. Gervasio, F. L. & Galdadas, I. Drug-resistant EGFR lung cancer mutations promote tumor growth by stabilizing interfaces in ligand-free signaling-competent EGFR oligomers. YARETA https://doi.org/10.26037/yareta:qtkuoibmhndc3jxcwtwzo7eeey (2024).

18. Schmitz, K. R., Bagchi, A., Roovers, R. C., Bergen, P. M. P. V. & Ferguson, K. M. Structural Evaluation of EGFR inhibition mechanisms for nanobodies/VHH domains. *Struct. Lond. Engl. 1993* **21**, 1214–1224 (2014).

19. Webb, B. & Sali, A. Comparative Protein Structure Modeling Using MODELLER. *Curr. Protoc. Bioinforma.* **54**, 5.6.1-5.6.37 (2016).

20. Garrett, T. P. J. *et al.* Crystal structure of a truncated epidermal growth factor receptor extracellular domain bound to transforming growth factor alpha. *Cell* **110**, 763–73 (2002).

21. Park, S.-J. *et al.* CHARMM-GUI Glycan Modeler for modeling and simulation of carbohydrates and glycoconjugates. *Glycobiology* **29**, 320–331 (2019).

22. Raman, E. P., Guvench, O. & MacKerell, A. D. CHARMM additive all-atom force field for glycosidic linkages in carbohydrates involving furanoses. *J. Phys. Chem. B* **114**, 12981–12994 (2010).

23. Warnock, D. E. *et al.* Determination of plasma membrane lipid mass and composition in cultured Chinese hamster ovary cells using high gradient magnetic affinity chromatography. *J. Biol. Chem.* **268**, 10145–10153 (1993).

24. Allouche, A.-R. Gabedit--a graphical user interface for computational chemistry softwares. *J. Comput. Chem.* **32**, 174–182 (2011).

25. Wu, E. L. *et al.* CHARMM-GUI Membrane Builder toward realistic biological membrane simulations. *J. Comput. Chem.* **35**, 1997–2004 (2014).

26. Huang, J. *et al.* CHARMM36m: An improved force field for folded and intrinsically disordered proteins. *Nat. Methods* **14**, 71–73 (2016).

27. Boonstra, S., Onck, P. R. & van der Giessen, E. CHARMM TIP3P Water Model Suppresses Peptide Folding by Solvating the Unfolded State. *J. Phys. Chem. B* **120**, 3692–3698 (2016).

28. Abraham, M. J. *et al.* GROMACS: High performance molecular simulations through multi-level parallelism from laptops to supercomputers. *SoftwareX* **1–2**, 19–25 (2015).

29. Bussi, G., Donadio, D. & Parrinello, M. Canonical sampling through velocity rescaling. *J. Chem. Phys.* **126**, 014101 (2007).

30. Parrinello, M. & Rahman, A. Polymorphic transitions in single crystals: A new molecular dynamics method. *J. Appl. Phys.* **52**, 7182–7190 (1981).

31. Hess, B., Bekker, H., Berendsen, H. J. C. & Fraaije, J. G. E. M. LINCS: A linear constraint solver for molecular simulations. *J. Comput. Chem.* **18**, 1463–1472 (1997).

32. Essmann, U. *et al.* A smooth particle mesh Ewald method. *J. Chem. Phys.* **103**, 8577–8593 (1995).

33. Lu, C. *et al.* Structural evidence for loose linkage between ligand binding and kinase activation in the epidermal growth factor receptor. *Mol. Cell. Biol.* **30**, 5432–43 (2010).
34. Endres, N. F. *et al.* Conformational coupling across the plasma membrane in activation of the EGF receptor. *Cell* **152**, 543–56 (2013).
35. Yasuda, H. *et al.* Structural, Biochemical, and Clinical Characterization of Epidermal Growth Factor Receptor (EGFR) Exon 20 Insertion Mutations in Lung Cancer. *Sci. Transl. Med.* **5**, 216ra177-216ra177 (2013).
36. Martínez-Rosell, G., Giorgino, T. & De Fabritiis, G. PlayMolecule ProteinPrepare: A Web Application for Protein Preparation for Molecular Dynamics Simulations. *J. Chem. Inf. Model.* **57**, 1511–1516 (2017).
37. Berendsen, H. J. C., Postma, J. P. M., van Gunsteren, W. F., Di Nola, A. & Haak, J. R. Molecular dynamics with coupling to an external bath. *J. Chem. Phys.* **81**, 3684–3690 (1984).
38. Zanetti-Domingues, L. C. *et al.* Determining the geometry of oligomers of the human epidermal growth factor family on cells with 7 nm resolution. *Prog. Biophys. Mol. Biol.* **118**, (2015).
39. Webb, S. E. D. *et al.* Nanometric molecular separation measurements by single molecule photobleaching. *Methods San Diego Calif* **88**, 76–80 (2015).
40. Needham, S. R. *et al.* Determining the geometry of oligomers of the human epidermal growth factor family on cells with <10 nm resolution. *Biochem. Soc. Trans.* **43**, (2015).
41. Needham, S. R. *et al.* Measuring EGFR Separations on Cells with ∼10 nm Resolution via Fluorophore Localization Imaging with Photobleaching. *PloS One* **8**, e62331 (2013).
42. Rolfe, D. J. *et al.* Automated multidimensional single molecule fluorescence microscopy feature detection and tracking. *Eur. Biophys. J. EBJ* **40**, 1167–86 (2011).
43. Garry, J., Li, Y., Shew, B., Gradinaru, C. C. & Rutenberg, A. D. Bayesian counting of photobleaching steps with physical priors. *J. Chem. Phys.* **152**, 024110 (2020).

44. Kiranyaz, S. *et al.* 1D convolutional neural networks and applications: A survey. *Mech. Syst. Signal Process.* **151**, 107398 (2021).

45. Wang, H. & Song, M. Ckmeans.1d.dp: Optimal k-means Clustering in One Dimension by Dynamic Programming. *R J.* **3**, 29–33 (2011).

46. Schuirmann, D. J. A comparison of the two one-sided tests procedure and the power approach for assessing the equivalence of average bioavailability. *J. Pharmacokinet. Biopharm.* **15**, 657–680 (1987).

47. Killick, R., Fearnhead, P. & Eckley, I. A. Optimal detection of changepoints with a linear computational cost. *J. Am. Stat. Assoc.* **107**, 1590–1598 (2012).

48. Pham-Gia, T. & Hung, T. L. The mean and median absolute deviations. *Math. Comput. Model.* **34**, 921–936 (2001).

49. Iglewicz, B. & Hoaglin, D. C. *How to Detect and Handle Outliers*. vol. 16 (ASQC Quality Press, Milwaukee, 1993).

50. Gelman, A. & Rubin, D. B. Inference from Iterative Simulation Using Multiple Sequences. *Stat. Sci.* **7**, 457–472 (1992).

51. Xue, J., Luo, Y. & Liang, F. Average (E)BIC-like Criteria for Bayesian Model Selection. 35 (2017).

52. Borg, I. & Groenen, P. J. *Modern Multidimensional Scaling: Theory and Applications*. (Springer Science & Business Media, 2005).

Uncropped Western Blots

Refers to Figure S1f

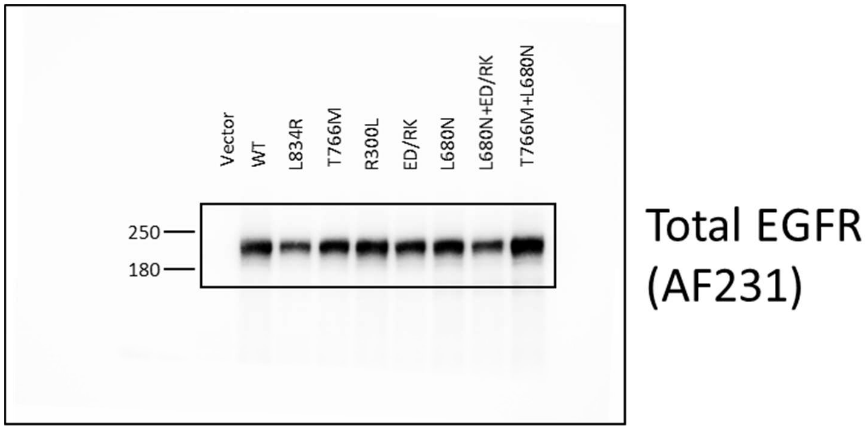

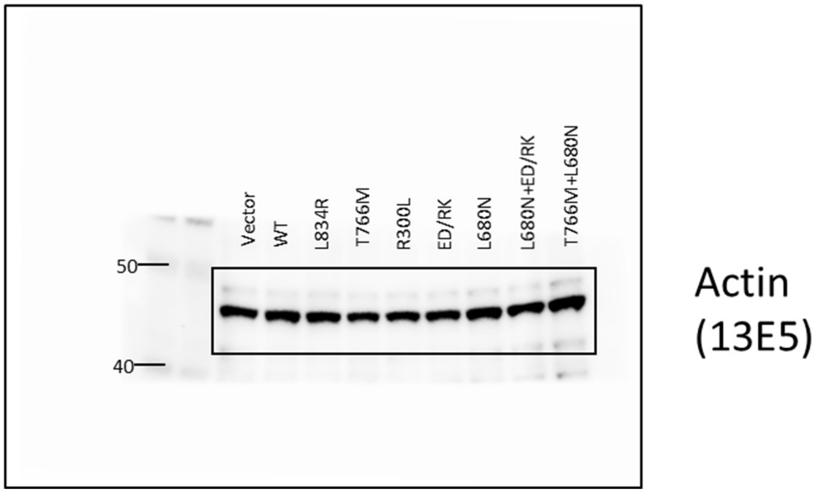

Refers to Fig. S3e

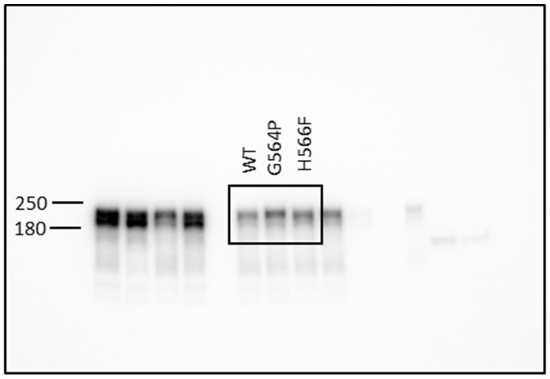

pY1068 EGFR
(D7A5)

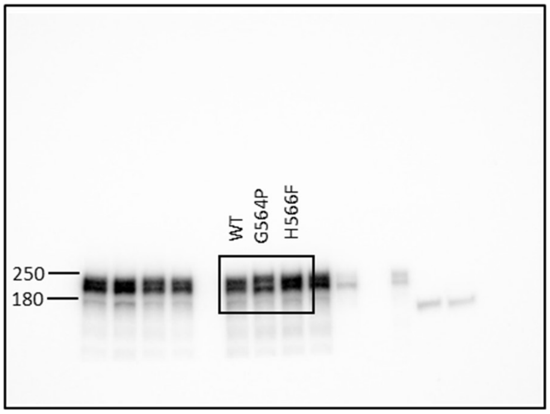

Total EGFR
(D38B1)

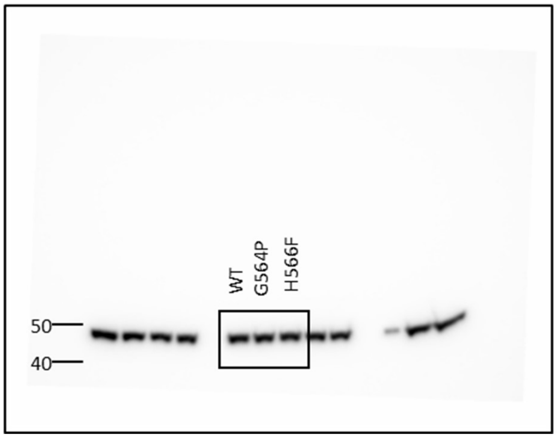

Actin
(13E5)

Refers to Fig. S7c

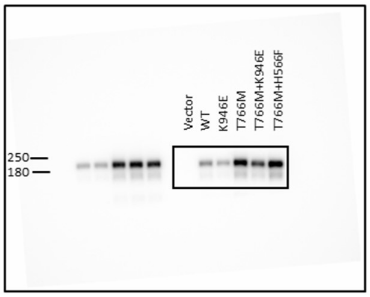

pY1068 EGFR (D7A5)

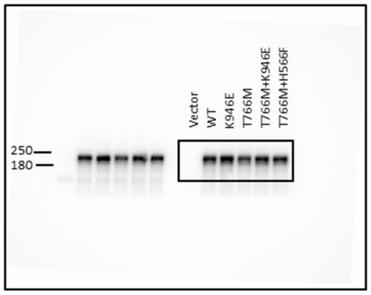

Total EGFR (AF231)

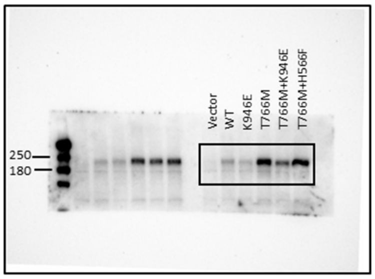

pY992 EGFR (EM12)

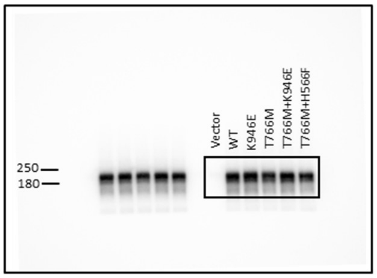

Total EGFR (D38B1)

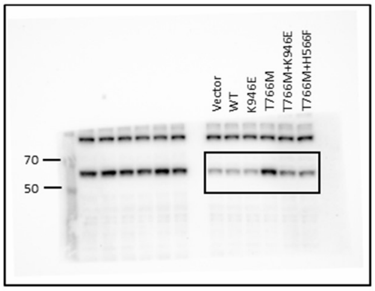

pS473 AKT (D9E)

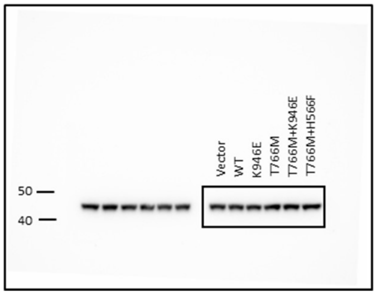

Actin (13E5)
