# Supplementary material for: Drug-resistant EGFR mutations promote lung cancer by stabilizing interfaces in ligand-free kinase-active EGFR oligomers
Source: Nat Commun. 2024 Mar 19;15:2130. doi: 10.1038/s41467-024-46284-x (PMC10951324; doi:10.1038/s41467-024-46284-x)
Supplement: Supplementary file 3 — Reporting Summary [file 41467_2024_46284_MOESM3_ESM.pdf]

nature portfolio

Corresponding author(s): Marisa L. Martin-Fernandez
Francesco L. Gervasio
Daniel J. Rolfe

Last updated by author(s): Jan 31, 2024

Reporting Summary

Nature Portfolio wishes to improve the reproducibility of the work that we publish. This form provides structure for consistency and transparency in reporting. For further information on Nature Portfolio policies, see our Editorial Policies and the Editorial Policy Checklist.

Statistics

For all statistical analyses, confirm that the following items are present in the figure legend, table legend, main text, or Methods section.

n/a	Confirmed	
☐	☒	The exact sample size (n) for each experimental group/condition, given as a discrete number and unit of measurement
☐	☒	A statement on whether measurements were taken from distinct samples or whether the same sample was measured repeatedly
☐	☒	The statistical test(s) used AND whether they are one- or two-sided<br>*Only common tests should be described solely by name; describe more complex techniques in the Methods section.*
☒	☐	A description of all covariates tested
☐	☒	A description of any assumptions or corrections, such as tests of normality and adjustment for multiple comparisons
☐	☒	A full description of the statistical parameters including central tendency (e.g. means) or other basic estimates (e.g. regression coefficient) AND variation (e.g. standard deviation) or associated estimates of uncertainty (e.g. confidence intervals)
☐	☒	For null hypothesis testing, the test statistic (e.g. F, t, r) with confidence intervals, effect sizes, degrees of freedom and P value noted<br>*Give P values as exact values whenever suitable.*
☐	☒	For Bayesian analysis, information on the choice of priors and Markov chain Monte Carlo settings
☒	☐	For hierarchical and complex designs, identification of the appropriate level for tests and full reporting of outcomes
☒	☐	Estimates of effect sizes (e.g. Cohen's d, Pearson's r), indicating how they were calculated

Our web collection on statistics for biologists contains articles on many of the points above.

Software and code

Policy information about availability of computer code

Data collection	NanoImager software Version: 1.7.3.10248<br>Zen Black 2.3, SP1 Zeiss RRID:SCR_018163
Data analysis	Phython 3.7 Python Software Foundation, https://www.python.org https://www.python.org; RRID:SCR_008394<br>Jupyter Notebook 6.4.1 https://github.com/jupyter/notebook RRID:SCR_018315<br>Pandas 1.3.5 https://github.com/pandas-dev/pandas https://github.com/pandas-dev/pandas; RRID:SCR_018214<br>MatPlotLib 3.5.1 https://github.com/matplotlib/matplotlib RRID:SCR_008624<br>Seaborn 0.11.2 https://github.com/mwaskom/seaborn RRID:SCR_018132<br>Scipy 1.7.3 https://github.com/scipy/scipy https://github.com/scipy/scipy; RRID:SCR_008058<br>Scikit-posthocs 0.6.7 http://github.com/maximtrp/scikit-posthocs RRID:SCR_021363<br>Phython 2.7 Python Software Foundation, https://www.python.org https://www.python.org; RRID:SCR_008394<br>R version 3.4.4 (2018-03-15) https://cran.r-project.org/bin/windows/base/old/ RRID:SCR_001905<br>Prism software version 9 GraphPad Software<br>https://www.graphpad.com RRID:SCR_002798<br>FIJI 2.9.0 (ImageJ 1.53t, Java 1.8.0_322) https://imagej.net/software/fiji/ RRID:SCR_003070<br>Trainable WEKA Segmentation plugin for FIJI v3.3.2 https://imagej.net/plugins/tws/ RRID:SCR_001214<br>Cellpose 2.0 https://www.cellpose.org RRID:SCR_021716<br>ImageLab Software Bio-Rad RRID:SCR_014210<br>Huygens Software 22.10 Scientific Volume Imaging RRID:SCR_014237<br><br>Due to the size, complexity and compute requirements for both we can provide access on request to raw FLImP or single particle tracking data

and code for this paper. The raw data analysed for this paper is ~300TB of single molecule movies. The code is a complex, multistage, automated batch-mode pipeline, requiring expertise to build, install and deploy. It is not designed for interactive use and requires significant compute resources to process a day's acquisition within ~1 day. To achieve this the pipeline is currently designed to be deployed on our local high-throughput compute cluster architecture comprising 10s of CPU and GPU workstations. The most realistic way to provide access to our code and data is to consider any requests individually, and identify in each case a practical way to achieve the needs of each request. Access to the FLImP technique and our deployed resources is available through peer-reviewed access to the Octopus facility (https://www.clf.stfc.ac.uk/Pages/Access-to-Octopus-and-Ultra.aspx).

For manuscripts utilizing custom algorithms or software that are central to the research but not yet described in published literature, software must be made available to editors and reviewers. We strongly encourage code deposition in a community repository (e.g. GitHub). See the Nature Portfolio guidelines for submitting code & software for further information.

Data

Policy information about availability of data

All manuscripts must include a data availability statement. This statement should provide the following information, where applicable:

- Accession codes, unique identifiers, or web links for publicly available datasets
- A description of any restrictions on data availability
- For clinical datasets or third party data, please ensure that the statement adheres to our policy

Access to primary and referenced "minimum datasets" for FLImP or single particle tracking will be shared by the lead contact upon request.
Source data are provided with this paper. The confocal data generated in this study to assess binding affinities have been deposited in the Zenodo database under accession code DOI: 10.5281/zenodo.10567248 and the input files for the MD simulations are deposited in YARETA under accession code DOI: 10.26037/yareta:qtkuoibmhndc3jxcwtwzo7eeey.

Research involving human participants, their data, or biological material

Policy information about studies with human participants or human data. See also policy information about sex, gender (identity/presentation), and sexual orientation and race, ethnicity and racism.

Reporting on sex and gender	This study did not involve human participants, their data nor biological material.
Reporting on race, ethnicity, or other socially relevant groupings	This study did not involve human participants, their data nor biological material.
Population characteristics	This study did not involve human participants, their data nor biological material.
Recruitment	This study did not involve human participants, their data nor biological material.
Ethics oversight	This study did not involve human participants, their data nor biological material.

Note that full information on the approval of the study protocol must also be provided in the manuscript.

Field-specific reporting

Please select the one below that is the best fit for your research. If you are not sure, read the appropriate sections before making your selection.

☒ Life sciences ☐ Behavioural & social sciences ☐ Ecological, evolutionary & environmental sciences

For a reference copy of the document with all sections, see nature.com/documents/nr-reporting-summary-flat.pdf

Life sciences study design

All studies must disclose on these points even when the disclosure is negative.

Sample size	For each condition, the 100 measurements with the highest resolution (smallest 69% confidence interval with a maximum separation measure of 8nm), acceptable isotropy (x/y >0.5) and with maximum separation no greater than 70nm were chosen for the FLImP fits. Except for the latter, these criteria did not bias the selection of separations representing each dataset. This is explained in supplementary note 3. The sample size for the single particle tracking data sets was greater than 3000 tracks per condition.
Data exclusions	Any measurement not in the top 100 was excluded from the FLImP fits.
Replication	All attempts of replication were successful. The acquisition of single particle tracking data was repeated 3 times per condition and each repeat generated ~1000 tracks. The western blots were repeated three times as is typical for these datasets. The growth curves in BA/F3 cells were repeated four times in line with previously published results. The number of mice assigned to each cohort was based on the statistics from the differences between the mutants expressed in the BA/F3 growth curves.
Randomization	The allocation of mice in the experiment was random.

Randomization | Samples within an experimental group were randomly allocated.

Blinding | Blinding was not necessary to our study as all measurements taken of the in vivo tumours were quantitative.
For tumour establishment it was necessary to know which cohort received what cell lines. Animal care and growth measurement staff was blind to the cohort details. Confirmatory histology for overall expression levels was done unblinded as tumours/tumour sizes were identifying the sections cut thereof by shape/size anyway.

Reporting for specific materials, systems and methods

We require information from authors about some types of materials, experimental systems and methods used in many studies. Here, indicate whether each material, system or method listed is relevant to your study. If you are not sure if a list item applies to your research, read the appropriate section before selecting a response.

Materials & experimental systems

n/a	Involved in the study
☐	☒ Antibodies
☐	☒ Eukaryotic cell lines
☒	☐ Palaeontology and archaeology
☐	☒ Animals and other organisms
☒	☐ Clinical data
☐	☐ Dual use research of concern
☒	☐ Plants

Methods

n/a	Involved in the study
☒	☐ ChIP-seq
☒	☐ Flow cytometry
☒	☐ MRI-based neuroimaging

Antibodies

Antibodies used

mAb 2E9 Abcam Cat# ab8465; RRID: AB_2096462, lot: GR125648-4
Anti-EGFR Affibody® Molecule Abcam Cat# ab95116; RRID:AB_11156238, lot GR37343-34
EGF Receptor (clone D38B1) XP Rabbit mAb EGFR Cell Signaling Technology Cat# 4267; RRID: AB_2246311, lot: 24
Phospho-Akt (Ser473) (clone D9E) XP® Rabbit monoclonal antibody Cell Signaling Technology Cat# 4060; RRID:AB_2315049, lot: 26
Phospho-EGF Receptor (Tyr1173) (clone 53A5) Rabbit mAb Cell Signaling Technology Cat# 4407; RRID: AB_331795, lot 6
Rabbit Anti-beta-Actin Monoclonal Antibody, HRP Conjugated, Clone 13E5 Cell Signaling Technology Cat# 5125; RRID: AB_1903890, lot 6
Phospho-EGF Receptor (Tyr1068) (clone D7A5) XP Rabbit mAb Cell Signaling Technology Cat# 3777; RRID: AB_2096270, lot: 13
Goat Human EGFR Affinity Purified Polyclonal Ab R & D Systems Cat# AF231; RRID: AB_355220 lot: AUC1219011
Mouse EGFR (phospho Y992) antibody [clone EM-12] Abcam Cat# ab81440; RRID: AB_1658463, lot: GR245205-6
Peroxidase Affinipure Donkey anti-mouse IgG (H+L) Jackson ImmunoResearch Europe Cat# 715-035-150; RRID:AB_2340770
Peroxidase Affinipure Donkey anti-rabbit IgG (H+L) Jackson ImmunoResearch Europe Cat# 711-035-152; RRID:AB_10015282
Peroxidase Affinipure Donkey anti goat IgG (H+L) Jackson ImmunoResearch Europe Cat# 705-035-147; RRID:AB_2313587

Validation

mAb 2E9 - Abpromise guarantee covers the use of ab8465 in Immunocytochemistry/Immunofluorescence and Flow Cytometry. References from Abcam's website: Moisan A et al. Inhibition of EGF Uptake by Nephrotoxic Antisense Drugs In Vitro and Implications for Preclinical Safety Profiling. Mol Ther Nucleic Acids 6:89-105 (2017). Ha SW et al. Ions doped melanin nanoparticle as a multiple imaging agent. J Nanobiotechnology 15:73 (2017). Zanetti-Domingues LC et al. Hydrophobic fluorescent probes introduce artifacts into single molecule tracking experiments due to non-specific binding. PLoS One 8:e74200 (2013). Defize LH et al. Signal transduction by epidermal growth factor occurs through the subclass of high affinity receptors. J Cell Biol 109:2495-507 (1989).

Anti-EGFR Affibody® - Validated for use in this study in our previous publications. Please see: Needham, S. R. et al. EGFR oligomerization organizes kinase-active dimers into competent signalling platforms. Nat. Commun. 7, 13307 (2016).
and Zanetti-Domingues, L. C. et al. The architecture of EGFR's basal complexes reveals autoinhibition mechanisms in dimers and oligomers. Nat. Commun. 9, 4325 (2018).

EGF Receptor (clone D38B1) XP Rabbit mAb EGFR is validated for use in Western Blotting, Immunoprecipitation, IHC Leica Bond, Immunohistochemistry, Immunofluorescence (Immunocytochemistry), Flow Cytometry. There are 1188 references on CST's website that cite this antibody, see: https://www.cellsignal.com/products/primary-antibodies/egf-receptor-d38b1-xp-rabbit-mab/4267

Phospho-Akt (Ser473) (clone D9E) XP® Rabbit monoclonal antibody is validated for use in Western Blotting, Immunoprecipitation, Immunohistochemistry, Immunofluorescence (Immunocytochemistry), Flow Cytometry. There are 11,001 references on CST's website that cite this antibody, see: https://www.cellsignal.com/products/primary-antibodies/phospho-akt-ser473-d9e-xp-rabbit-mab/4060

Phospho-EGF Receptor (Tyr1173) (clone 53A5) Rabbit mAb is validated for use in Western Blotting, Immunoprecipitation, Immunohistochemistry. There are 246 references on CST's website that cite this antibody, see: https://www.cellsignal.com/products/primary-antibodies/phospho-egf-receptor-tyr1173-53a5-rabbit-mab/4407

Rabbit Anti-beta-Actin Monoclonal Antibody (HRP Conjugated) Clone 13E5 is validated for use in Western Blotting. There are 502 references on CST's website that cite this antibody, see: https://www.cellsignal.com/products/antibody-conjugates/b-actin-13e5-

rabbit-mab-hrp-conjugate/5125

Phospho-EGF Receptor (Tyr1068) (clone D7A5) XP Rabbit mAb is validated for use in Western Blotting, Immunoprecipitation, Immunohistochemistry, Flow Cytometry. There are 888 references on CST's website that cite this antibody, see:https://www.cellsignal.com/products/primary-antibodies/phospho-egf-receptor-tyr1068-d7a5-xp-rabbit-mab/3777

Goat Human EGFR Affinity Purified Polyclonal Ab is validated for use in Western Blotting, Immunohistochemistry, Flow Cytometry. There are 15 references on RnD system's website that cite this antibody, see: https://www.rndsystems.com/products/human-egfr-antibody_af231#product-citations

Mouse EGFR (phospho Y992) antibody [clone EM-12] Abpromise guarantee covers the use of ab81440 in Western Blotting, Immunoprecipitation. This antibody has been cited in: Deng QF et al. Cyclooxygenase-2 mediates gefitinib resistance in non-small cell lung cancer through the EGFR/PI3K/AKT axis. J Cancer 11:3667-3674 (2020). Hu DD et al. SKA3 promotes lung adenocarcinoma metastasis through the EGFR-PI3K-Akt axis. Biosci Rep 40:N/A (2020). Pei YF et al. Silencing of LAMC2 Reverses Epithelial-Mesenchymal Transition and Inhibits Angiogenesis in Cholangiocarcinoma via Inactivation of the Epidermal Growth Factor Receptor Signaling Pathway. Am J Pathol 189:1637-1653 (2019). Dittmann K et al. New roles for nuclear EGFR in regulating the stability and translation of mRNAs associated with VEGF signaling. PLoS One 12:e0189087 (2017). Needham SR et al. EGFR oligomerization organizes kinase-active dimers into competent signalling platforms. Nat Commun 7:13307 (2016). Harrison H et al. Oestrogen increases the activity of oestrogen receptor negative breast cancer stem cells through paracrine EGFR and Notch signalling. Breast Cancer Res 15:R21 (2013).

Peroxidase Affinipure Donkey anti-mouse IgG (H+L) is validated as: Target: Mouse, Host: Donkey, Antibody Format: Whole IgG Specificity: IgG (H+L), Minimal Cross Reactivity: Bovine, Chicken, Goat, Guinea Pig, Syrian Hamster, Horse, Human, Rabbit, Sheep Serum Proteins, Conjugate: Horseradish Peroxidase. There are 669 references on Jackson Immunoresearch's website that cite this antibody, see: https://www.jacksonimmuno.com/catalog/products/715-035-150

Peroxidase Affinipure Donkey anti-rabbit IgG (H+L) is validated as: Target: Rabbit, Host: Donkey, Antibody Format: Whole IgG Specificity: IgG (H+L), Minimal Cross Reactivity: Bovine, Chicken, Goat, Guinea Pig, Syrian Hamster, Horse, Human, Mouse, Rat, Sheep Serum Proteins, Conjugate: Horseradish Peroxidase. There are 1073 references on Jackson Immunoresearch's website that cite this antibody, see: https://www.jacksonimmuno.com/catalog/products/711-035-152

Peroxidase Affinipure Donkey anti goat IgG (H+L) is validated as: Target: Goat, Host: Donkey, Antibody Format: Whole IgG Specificity: IgG (H+L), Minimal Cross Reactivity: Chicken, Guinea Pig, Syrian Hamster, Horse, Human, Mouse, Rabbit, Rat Serum Proteins, Conjugate: Horseradish Peroxidase. There are 231 references on Jackson Immunoresearch's website that cite this antibody, see: https://www.jacksonimmuno.com/catalog/products/705-035-147

Eukaryotic cell lines

Policy information about cell lines and Sex and Gender in Research

Cell line source(s)	Hamster: CHO cells, gift from Prof. Peter Parker (The Francis Crick Institute, UK)<br>Ba/F3 Creative Biogene Cat# CSC-C2045<br>mAb108 expressing hybridoma cells ATCC Cat# HB-9764
Authentication	None of the cell lines used were authenticated after receipt from the suppliers
Mycoplasma contamination	All cell lines tested negative for mycoplasma
Commonly misidentified lines<br>(See ICLAC register)	No misidentified cell lines were used

Animals and other research organisms

Policy information about studies involving animals; ARRIVE guidelines recommended for reporting animal research, and Sex and Gender in Research

Laboratory animals	In this study, young adult (6-7 weeks old, 24.6±2.1 g) male NOD.Cg-Prkdcscid Il2rgtm1Wjl/SzJ mice (NSG; purchased from Charles River UK, strain code 614, RRID:IMSR_JAX005557) were used for all animal experiments.<br>Mice were maintained within the King's College London Biological Services Unit under specific pathogen-free conditions in a dedicated and licensed air-conditioned animal room (at 23±2°C and 40-60% relative humidity) under light/dark cycles lasting 12h every day. They were kept in individually ventilated standard plastic cages (501cm2 floor space; from Tecniplast) including environmental enrichment and bedding material in the form of sterilized wood chips, paper stripes and one cardboard roll per cage. Maximum cage occupancy was five animals, and animals were moved to fresh cages with fresh environmental enrichment and bedding material twice per week. Sterilized tap water and food were available ad libitum; food was PicoLab Rodent Diet 20 (LabDiet) in the form of 2.5x1.6x1.0 cm oval pellets that were supplied at the top of the cages.
Wild animals	The study did not involve wild animals
Reporting on sex	Findings only apply to male mice and we considered males in study design because lung cancer has a higher incidence in human males
Field-collected samples	The study did not involve samples collected from the field

Ethics oversight	All experimental protocols were monitored and approved by the King's College London Animal Welfare and Ethical Review Body in accordance with UK Home Office regulations (PPL PP4067431) under the Animals (Scientific Procedures) Act 1986 and UK National Cancer Research Institute (NCRI) Guidelines for the Welfare and Use of Animals in Cancer Research.

Note that full information on the approval of the study protocol must also be provided in the manuscript.
